# What do macroinvertebrate indices measure? Stressor‐specific stream macroinvertebrate indices can be confounded by other stressors

**DOI:** 10.1111/fwb.14106

**Published:** 2023-05-17

**Authors:** J. Iwan Jones, Charlotte E. M. Lloyd, John F. Murphy, Amanda Arnold, Chas P. Duerdoth, Adrianna Hawczak, James L. Pretty, Penny J. Johnes, Jim E. Freer, Moragh W. Stirling, Carla Richmond, Adrian L. Collins

**Affiliations:** ^1^ School of Biological and Behavioural Sciences Queen Mary University of London London UK; ^2^ School of Geographical Sciences University of Bristol Bristol UK; ^3^ Cabot Institute University of Bristol Bristol UK; ^4^ School of Archaeology, Geography and Environmental Sciences University of Reading Reading UK; ^5^ Soils, Agriculture and Water, RSK ADAS Ltd Chester UK; ^6^ Net Zero and Resilient Farming, Rothamsted Research Okehampton UK

**Keywords:** bioassessment, diffuse pollution, high frequency monitoring, hydrochemistry, multiple stressors

## Abstract

1. Monitoring programmes worldwide use biota to assess the “health” of water bodies. Indices based on biota are used to describe the change in status of sites over time, to identify progress against management targets and to diagnose the causes of biological degradation. A variety of numerical stressor‐specific biotic indices have been developed based on the response of biota to differences in stressors among sites. Yet, it is not clear how variation in pressures within sites, over what time period, and in what combination has the greatest impact on different biotic groups. An understanding of how temporal variation in pressures influences biological assessment indices would assist in setting achievable targets and help focus catchment‐scale mitigation strategies to ensure that they deliver the desired improvements in biological condition.
2. Hydrochemical data provided by a network of high‐frequency (15 or 30 min) automated monitoring stations over 3 years were matched to replicated biological data to understand the influence of spatio‐temporal variation in pollution pressures on biological indices. Hydrochemical data were summarised in various ways to reflect central tendency, peaks, troughs and variation over 1–90 days before the collection of each biological sample. An objective model selection procedure was used to determine which hydrochemical determinand, and over what time period, best explained variation in the biological indices.
3. Stressor‐specific indices derived from macroinvertebrates which purportedly assess stress from low flows, excess fine sediment, nutrient enrichment, pesticides and organic pollution were significantly inter‐correlated and reflected periods of low oxygen concentration, even though only one index (ASPT_WHPT_, average score per taxon) was designed for this purpose. Changes in community composition resulting from one stressor frequently lead to confounding effects on stressor‐specific indices.
4. Variation in ASPT_WHPT_ was best described by dissolved oxygen calculated as Q_5_ over 10 days, suggesting that low oxygen events had most influence over this period. Longer‐term effects were apparent, but were masked by recovery. Macroinvertebrate abundance was best described by Q_95_ of stream velocity over 60 days, suggesting a slower recovery in numbers than in the community trait reflected by ASPT_WHPT_.
5. Although use of ASPT_WHPT_ was supported, we recommend that additional independent evidence should be used to corroborate any conclusions regarding the causes of degradation drawn from the other stressor‐specific indices. The use of such stressor‐specific indices alone risks the mistargeting of management strategies if the putative stressor‐index approach is taken to be more reliable than the results herein suggest.

## 1 INTRODUCTION

As biota can provide an integrated overview of prevailing conditions and “health” of a water body (Furse et al., 2006; Hawkins et al., 2010), biological assessment often is used as a measure of the condition of freshwater resources, to diagnose causes of biological degradation, to describe change in status over time, and to identify progress against management targets (Birk et al., 2012; Jones et al., 2010). In addition to providing an integrated assessment of ecosystem condition, biota offers other potential advantages. Many pollutants, particularly those arising from diffuse sources, are delivered to watercourses as episodic events, often associated with peaks in precipitation (e.g., storms; Kronvang et al., 1997; Ng et al., 1993; Ockenden et al., 2017). Whereas detection and characterisation of these episodic events may require intensive physical and chemical sampling through time (Johnes, 2007; Lloyd et al., 2014), such events can cause lasting changes to biotic communities such that a single biological sample can reflect stress over a substantial time period: one invertebrate sample is capable of determining mean pH with a precision comparable to a year of fortnightly direct measurements of water chemistry (Ormerod et al., 2006). In recognition of their utility, biological assessments of the condition of fresh waters are now included in many monitoring programmes worldwide (Nichols et al., 2017) and form a central pillar of the European Union's Water Framework Directive (WFD: European Parliament, 2000) where assessment based on different biological quality elements are combined to provide an overall ecological status.

Working from a premise that sensitivities to stressors vary among species and biotic groups, a variety of numerical stressor‐specific biotic indices have been developed and are used to aid interpretation of the causes of biological degradation of fresh waters. For some time, indices have been based on perceived tolerance of organisms to pollution as assessed by expert judgement, such as ASPT, LIFE, PSI, SPEAR (Armitage et al., 1983; Beketov et al., 2009; Extence et al., 1999, 2013: for details of all biological indices see Table S1), but latterly statistical approaches have been used in conjunction with empirical data to improve these indices (MCI, WHPT, ePSI; Clapcott et al., 2017; Paisley et al., 2014; Turley et al., 2016; Turley et al., 2015) and to develop new indices a priori (Sed‐MCI, AWIC, CoFSI, BSTI, TRPI; Clapcott et al., 2017; Everall et al., 2019; Hubler et al., 2016; Murphy et al., 2013; Murphy et al., 2015). Now indices are available for a wide range of pressures, including among others, organic pollution, eutrophication, acidification, pesticides, excess fine sediment and flow pressures, as well as general degradation (Birk et al., 2012).

The more robust of these indices are based on objective statistical approaches, rather than a presumed knowledge of the underlying causal mechanisms, which can be flawed (Jones et al., 2017) or confounded by other factors at the field scale (Demars & Edwards, 2009; Jones et al., 2012). Nevertheless, species do respond to a variety of environmental and biological parameters. It is now recognised that multiple stressors frequently act in concert on fresh waters shaping ecological responses (Birk et al., 2020), where the response to one stressor can be conditioned by the response to another stressor operating in the same environmental space.

Multiple stressors can influence biological communities in three ways by (Vinebrooke et al., 2004):

1. having an interacting effect on the proximal driver of biological change,
2. affecting the sensitivity of biota to additional stressors, or
3. altering the biological community such that the expected response is compromised.

Hence, there is the potential for variation in environmental drivers, both natural and anthropogenic, other than the pressure of interest to cause the returned values of biotic indices to vary (Murphy & Davy‐Bowker, 2005).

Furthermore, the development of indices typically has relied on comparison of biological communities among sites along gradients corresponding to the specific pressure of interest (e.g., Clapcott et al., 2017; Murphy et al., 2013; Paisley et al., 2014; Turley et al., 2015). As drivers of environmental degradation cause change to a range of environmental conditions, it is often not clear to which pressure the biota are responding. For example, organic pollution causes change to concentrations of oxygen by driving microbial metabolism, which changes the redox status of the water column and sediment, leading to an increase in the proportion of free ammonia (NH_3_‐N) and release of sediment‐bound phosphorous (P) to the water column, as well as changes in the physical character of sediment (Hynes, 1960; Jones et al., 2008). Establishing which of these pressures are driving the biological response is a challenge: true causal relationships can only be established in controlled manipulative experiments. As a consequence, rather than establishing the determinand that is directly responsible for driving change, it is often those determinands which are easy to measure that are used when assuming relationships with biota (Turley et al., 2016). Whilst establishing exact causal mechanisms may appear irrelevant for management (e.g., a change in the extent of organic pollution will produce a response in the index, irrespective of the mechanism), because most sites are exposed to multiple pressures, a clear understanding of causal mechanisms can aid in improved interpretation of biological change and, thus, identification of the source of problems, ultimately assisting in the targeting of mitigation efforts.

A related issue is the temporal variation in pressures. Stream hydrochemistry is a function of many interacting variables and processes, each operating at a range of temporal and spatial scales (Heathwaite et al., 1996). At the catchment scale, delivery of pollutants from both point and diffuse sources, combined with in‐river transformations, drive both short‐ and longer‐term trends in water quality (Lloyd et al., 2016, 2016b). Much of the development and testing of biological indices has been based on a principle of space‐for‐time substitution, where the response to variation in pressure among spatially distinct sites is assumed to replicate the response to variation in the pressure within individual sites. To achieve this, within‐site temporal variation in hydrochemical datasets has been reduced to some summary measure (typically mean or return period). Yet, in many cases, it is not clear what aspect of variation in pressures (e.g., peaks, troughs, central tendency or variation) has the greatest impact on biota. As the various mitigation strategies available to combat pollution address different aspects of the delivery of potential pollutants to rivers, the mitigation strategy adopted (in terms of the options or combination of options chosen and their spatial configuration) will affect different parts of the variation in hydrochemistry. An understanding of how temporal variation in pressures influences biological responses would help focus mitigation strategies at the catchment scale, to address how pollutants are delivered to watercourses in the most cost‐effective manner to deliver the desired improvements in biological condition.

The recent expansion of the use of *in situ* sensors to monitor hydrochemical determinands routinely at high temporal resolution is making detailed analysis of catchment behaviours more feasible, even in extreme environments (Blaen et al., 2016). Traditionally, few determinands (e.g., turbidity) have been measured at high frequency and used as a surrogate for the transport of other contaminants mobilised by peak flows but which cannot be measured directly with existing sensor technologies (Grayson et al., 1996; Kronvang et al., 1997; Stubblefield et al., 2007). The more recent introduction of novel sensor systems and bankside, automated photometers means that determinands such as nitrate‐nitrogen (NO_3_‐N) and total P (TP) can now be measured at higher temporal resolutions than previously possible (Blaen et al., 2016; Jarvie et al., 2018; Mellander et al., 2012). These high‐frequency measurements give more realistic estimates of the true variation in ecologically relevant determinands, such as sediment, nutrients and organic matter and, hence, better estimates of pollutant loads (Lloyd et al., 2016) and a greater understanding of the catchment controls on delivery of pollutants (Lloyd et al., 2016b). These high‐frequency data further open the opportunity to explore the influence of temporal variation in diffuse pollution pressures on biological responses, which in turn can provide a basis for assessing biotic indices. Whilst a variety of numerical stressor‐specific biotic indices have been developed, it is not clear how variation in pressures within sites, over what time period, and in what combination has the greatest impact on different biotic groups. An understanding of how temporal variation in pressures influences biological assessment indices would assist in setting achievable targets and help focus catchment‐scale mitigation strategies to ensure that they deliver the desired improvements in biological condition. Here we use data provided by a network of matched high‐frequency automated hydrochemical monitoring stations to understand better the response of biological communities to spatio‐temporal variation in pollution pressures. Working from the hypothesis that the biotic indices reflect the pressures they are designed to assess, we address two predictions, (i) biotic indices can be used to interpret change in biological communities to independently assess multiple different stressors relevant to management, (ii) biotic index values reflect episodic events, where the resilience of communities influences thetime over which such events are represented. To address these questions, we compared high‐frequency hydrochemical data, summarised in various ways and over different time periods, with simultaneous but statistically independent biological data, summarised as biotic indices, collected from sites subject to a range of diffuse and point‐source pressures. Specifically, we were interested in, and what aspect of the variation in, hydrochemical determinands (peaks, troughs, central tendency or variation) were most strongly correlated with variation in biotic indices and over what antecedent time periods.

## 2 MATERIALS AND METHODS

### 2.1 Site descriptions

**FIGURE 1:**
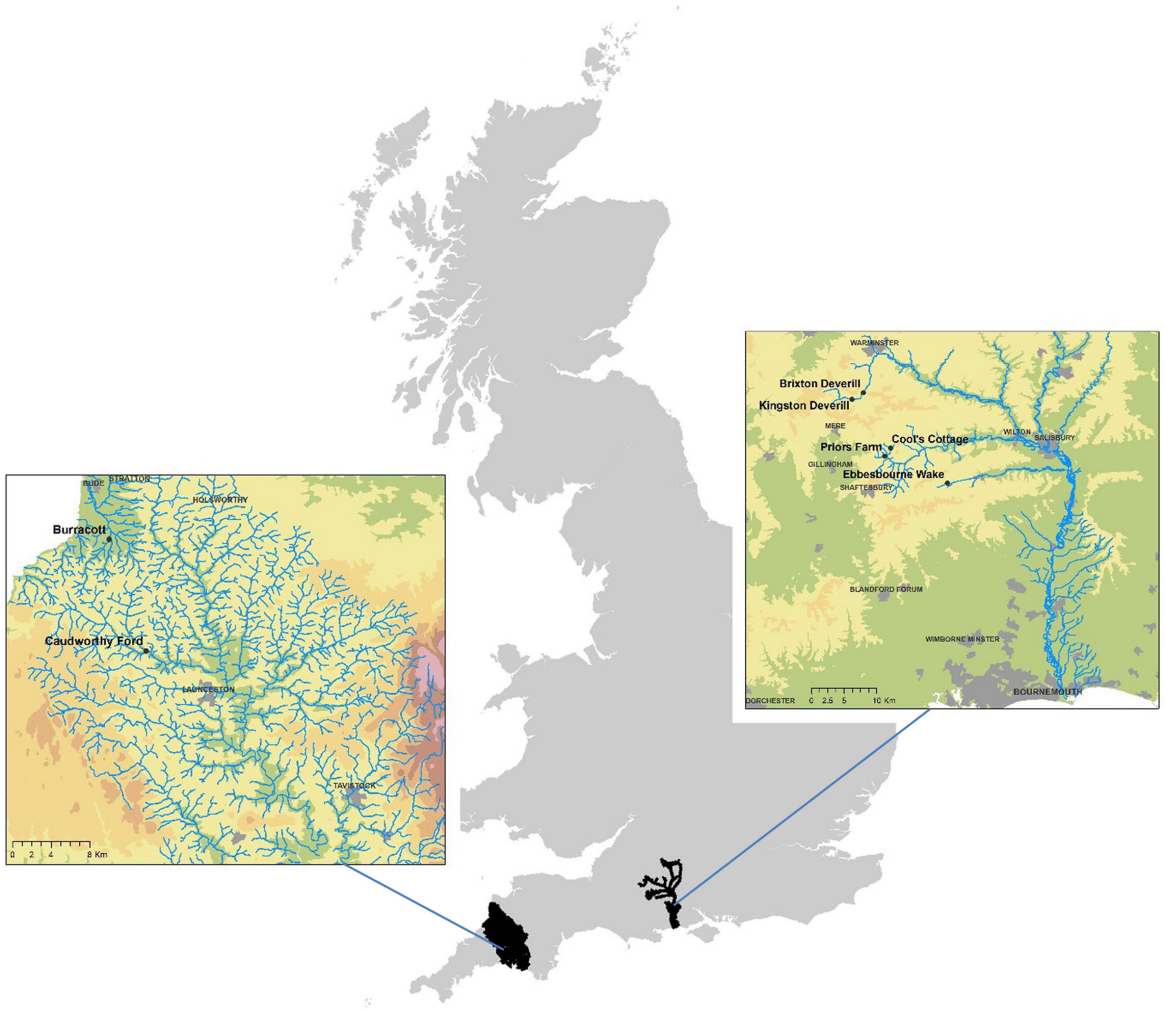
Location of sites.

The sub‐catchments studied are located in the headwaters of the Hampshire Avon, Tamar and Neet in southern England (Figure 1), which are predominantly agricultural with a mix of semi‐natural woodland, pasture (rough and improved) and arable land use (Zhang et al., 2012). These catchments comprise the study sites for the Hampshire Avon Demonstration Test Catchment (DTC) Project (http://www.avondtc.org.uk/Home.aspx) as part of the national DTC programme, designed to gather empirical evidence on the cost‐effectiveness of combinations of diffuse pollution mitigation measures at catchment scales, and funded by the UK Department for Environment, Food and Rural Affairs (Defra). The sub‐catchments were chosen to represent fresh waters with contrasting character and land use (Table 1), following a paired experimental design, where multiple on‐farm mitigation measures were implemented in one of each pair after a control period of business as usual (McGonigle et al., 2014). Spatially matched biological and hydrochemical monitoring stations were established at the outlet of each sub‐catchment, and water quality and biology monitored from spring 2010 to autumn 2013.

**TABLE 1:** Sub‐catchment characteristics of the sites examined.

Catchment	Hampshire Avon					Tamar	
River	Sem		Ebble	Wylye		Caudworthy water	Neet
Monitored location	Cool's cottage	Priors farm	Ebbesbourne wake	Kingston Deverill	Brixton Deverill	Caudworthy Ford	Burracott
Area (km^2^)^a^	2.6	4.6	16.7	25.2	50.2	16.6	10.9
Average rainfall (mm)^a^	897	863	912	980	967	1137	1067
Base Flow Index (BFI; ratio)^a^	0.49	0.23	0.97	0.89	0.93	0.35	0.44
Monitored elevation (m above sea level)^a^	163	126	165	190	189	139	108
Average slope (%)^a^	10.1	2.7	12.9	6.72	7.7	5.4	11.2
Dominant geology	Jurassic mudstone with clay		Cretaceous chalk with Upper Greensand			Carboniferous mudstone, siltstone, sandstone	
Soil types	Slightly acid loams and clays		Shallow limey loams with slightly acid loams and clays			Slightly acid loams and clays	
Dominant land use	Intensive dairy farming	Intensive dairy farming	Mixed arable farming	Mixed arable farming	Mixed arable farming	Intensive beef cattle farming	Intensive beef cattle farming
Arable (%)^b^	14	0	20	40	49	13	11
Improved pasture (%)^b^	37	77	52	36	30	73	77
Rough grazing (%)^b^	9	14	11	12	11	0	0
Woodland (%)^b^	38	6	5	4	3	10	10
Urban (%)^b^	2	3	12	8	7	4	2
WFD (2012) Classification	Moderate	Moderate	Poor	Poor	Moderate	Moderate	Moderate

^a^ Based on the Flood Estimation Handbook (Robson & Reed, 1999).

^b^ Based on the ADAS land use database and for reference year 2010.

### 2.2 High frequency hydrochemistry data

In each sub‐catchment, discharge was calculated from stage height and velocity logged at 15‐min intervals (for full details, see Lloyd et al., 2019). Electrical conductivity, pH, dissolved oxygen (DO), temperature, turbidity, and ammonium (NH_4_‐N) and chlorophyll‐*a* concentrations were determined at 15‐min intervals with a YSI 6600 V2 sonde (Sontek/YSI Inc.) mounted in a flow‐through reservoir. The probes were cleaned and calibrated once a month to reduce instrument malfunction and drift. Un‐ionised ammonia (NH_3_‐N) concentrations were determined by calculation from measured NH_4_‐N concentrations, pH and temperature (Emerson et al., 1975).

Nutrient concentrations (TP, total reactive phosphorus [TRP] and NO_3_‐N), were determined *in situ*. A flow‐through reservoir was refreshed at 30‐min intervals, from which samples were drawn for TP and TRP analyses using a wet chemistry analyser (Hach Lange Phosphax Sigma) which measures TP and TRP alternately using a colourimetric molybdate method by acid phase digestion performed at high temperature and pressure, with the digestion omitted on the TRP cycle. Each analysis takes approximately 10 min. Nitrate was determined using a UV optical sensor (Hach Lange Nitratax Plus SC), which was calibrated every 3 months as recommended by the manufacturer. The instruments were automatically calibrated once a day and the reagents were renewed once every 3 months, again in line with the recommendations of the manufacturer. This resulted in a 30‐min resolution dataset for nutrients.

In addition, a pair of ISCO 3700 samplers were installed at each site for automated water sample collection at daily (sampler 1) and sub‐daily (sampler 2) timescales. Samples collected by auto‐samplers were collected once per week and analysed in the laboratory to determine dissolved organic carbon (DOC: measured as non‐purgeable organic carbon [NPOC] following the methods outlined in Yates et al., 2016), N species (total N [TN], total dissolved N [TDN], nitrate, total ammoniacal N [NH_4_‐N]), and P fractions (total dissolved P [TDP], soluble reactive P [SRP] and TP). Other particulate, dissolved organic and molybdate‐unreactive fractions were calculated from these as follows:

- TN–TDN = particulate N (PN)
- TDN–nitrate–ammonium = dissolved organic N (DON)
- TDP–SRP = dissolved organic P (DOP)

The uncertainties associated with bank‐side analysis versus quality controlled laboratory analysis of daily auto‐sampler samples, with different sampling frequencies, and the data streams generated, in terms of the ability to detect change in hydrochemical time series, have been dealt with elsewhere (Lloyd et al., 2014, 2016, 2016a).

### 2.3 Biological data

Samples of macroinvertebrates were collected from sites matched to those used for hydrochemical monitoring (immediately downstream) using a semi‐quantitative (fixed effort) kick sample representative of the reach, following the WFD compliant standard RIVPACS methodology (3 min kick sample covering all habitats in proportion to their occurrence plus 1 min search), and preserved. Two such independent samples were collected from undisturbed sections of each site in spring, summer and autumn of each year (2010–2013), working in an upstream manner, and processed separately. The whole sample was processed where the abundance of each taxon present in the sample, resolved to mixed taxonomic (largely species) level, was determined upon return to the laboratory.

### 2.4 Data analysis

**FIGURE 2:**
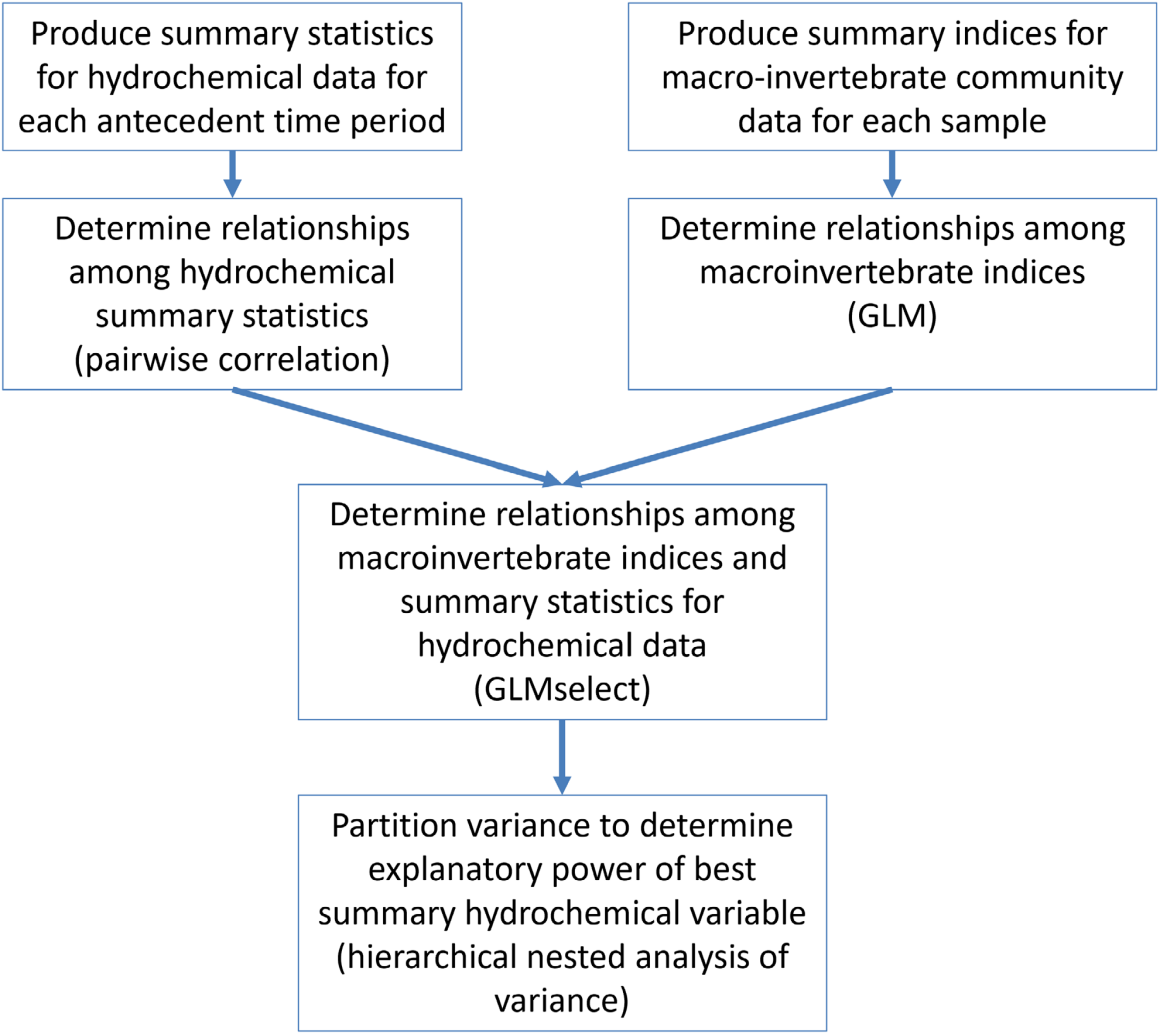
Schematic illustrating sequence of analyses undertaken.

The first step was to summarise the hydrochemical data to characterise aspects of the variation over various time periods (Figure 2). Measures of central tendency (mean, median), peaks (Q_90_, Q_95_, maximum, number of days exceeding 3 × Q_50_), troughs (minimum, Q_5_, Q_10_) and variation (coefficient of variation, range encompassing 50%, 80% and 90% of values) in the hydrochemical data were calculated over a range of antecedent time periods (1, 5, 10, 20, 30, 60 and 90 days) before the collection of the biological samples (for summary, see Table S2). The time periods were selected on the assumption that the increasing time range would capture those events that had the most relevance, starting from the “here‐now” (the day the biological sample was collected) and extending to the previous biological sampling occasion (and not further to avoid overlap and, hence, a lack of independence among data points). Although the number of measurements used to establish the summary statistics varied with the length of the antecedent period (from 96 to 9,504), all summary statistics were given equal weight in the analysis.

In order to establish relationships among the hydrochemical summary statistics (see Figure 2), Pearson correlations among pairs of summary statistics were undertaken both within determinands across all time periods (Figure S1), and within time periods across all determinands (Figure S2). This first step was critical to understand the independent explanatory power of each summary statistic describing the hydrochemical variables.

The biological data derived from each sample were summarised as biotic indices based on community composition. These comprised the abundance weighted versions of the WHPT (Whalley, Hawkes, Paisley and Trigg; Paisley et al., 2014) indices NTAXA_WHPT_ (number of WHPT scoring taxa) and ASPT_WHPT_ (average WHPT score per taxon), LIFE (Lotic‐invertebrate Index for Flow Evaluation; Extence et al., 1999), PSI (Proportion of Sediment‐sensitive Invertebrates; Extence et al., 2013), ePSI (empirically weighted Proportion of Sediment‐sensitive Invertebrates; Turley et al., 2016), CoFSI (Combined Fine Sediment Index; Murphy et al., 2015), TRPI (Total Reactive Phosphorus Index; Everall et al., 2019), SPEAR_pesticides_ (SPEcies At Risk of pesticides; Beketov et al., 2009), and log_10_ Abundance of all invertebrates. (For details of all indices and the putative stressors that they assess, see Table S1.) Similar to the hydrochemical data, the inter‐relatedness among biotic indices was established using generalised linear models, to understand the relationships both within and among sites (i.e., incorporating temporal variation at individual sites and spatial variation among sites, respectively; Table S3).

Once correlations among both the potential drivers (hydrochemical summary statistics) and responses (invertebrate indices) had been established (see Table 2; Figures S1 and S2), the GLMSELECT selection procedure in SAS/STAT® (SAS Institute Inc.) was used to determine which hydrochemical summary statistics, and over what time period, best described variation in the biotic indices (Figure 2). Whilst the approach used is robust to deviations from linearity, the biological indices have been developed to provide a linear (or near linear) response to the putative stressors being assessed (Murphy et al., 2015; Paisley et al., 2014; Turley et al., 2015). The model selection procedure enabled the interrelatedness of data collected from individual sites over multiple occasions, with two random samples nested within sites and occasions, to be correctly accounted for in the covariance structure. The complete set of hydrochemical determinands used comprised discharge, velocity, turbidity, % DO, pH, and concentrations of TP, SRP, DOP, TN, NO_3_‐N, total NH_4_‐N, free NH_3_‐N, DON, particulate organic nitrogen (PON) and DOC. All summary hydrochemical variables were offered for selection, together with site, time and sample (nested within site and occasion; Table S3), and significant best‐fit models selected in a stepwise manner using the Schwarz Bayesian information criterion (Judge et al., 1985; Schwarz, 1978).

**TABLE 2:** Average of pairwise correlations among summary statistics of hydrochemical determinands. For full details of all pairwise comparisons see Figures S1 and S2.

	Discharge	Velocity	Turbidity	pH	DO	TP	SRP	DOP	TN	DON	NO_3_‐N	NH_4_‐N	Free NH_3_‐N	PON
DOC	0.03	0.108	0.260	−0.012	−0.083	0.353	0.236	0.293	0.012	0.165	−0.102	0.237	0.164	0.121
PON	−0.044	−0.047	0.030	0.071	−0.102	0.278	0.029	0.068	0.196	0.070	0.039	0.072	0.103	
Free NH_3_‐N	−0.138	−0.096	0.039	0.224	−0.032	0.172	0.171	0.054	0.175	0.084	0.103	0.355		
NH_4_‐N	0.023	0.084	0.181	−0.062	−0.001	0.162	0.118	0.059	0.128	0.071	0.053			
NO_3_‐N	−0.011	−0.069	−0.085	0.188	−0.009	0.021	0.152	−0.047	0.334	0.091				
DON	−0.028	−0.037	0.067	0.101	−0.003	0.147	0.077	0.193	0.167					
TN	−0.043	−0.076	−0.032	0.189	−0.020	0.187	0.134	0.034						
DOP	−0.031	0.022	0.115	0.006	−0.041	0.213	0.116							
SRP	−0.088	−0.034	0.088	0.182	−0.059	0.324								
TP	−0.093	−0.035	0.138	0.084	−0.139									
DO	0.012	−0.012	−0.098	0.022										
pH	−0.199	−0.158	−0.082											
Turbidity	0.131	0.207												
Velocity	0.346													

Once best‐fit models of hydrochemical summary variables had been identified, the influence of the duration of the antecedent temporal period on the strength of the relationship between the summary variable and biotic response was determined. A hierarchical nested ANOVA (Table S3) was then used to partition the variance in order to determine the relative contribution of the summary hydrochemical variable, compared with that attributable to temporal and spatial variation, and other unknown sources of variation.

## 3 RESULTS

As expected, there was considerable correlation within summary measures of individual chemical determinands over a range of antecedent time periods (Figure S1), as well as correlation among determinands (Table 2; Figure S2). Over longer time periods, the summary hydrochemical variables became more stable and, therefore, more correlated among individual determinands (Figure S1) and between determinands (Figure S2). The number of days at three times Q_50_ was frequently unrelated to other summary statistics, particularly over shorter time periods (Figure S1), owing to a lack of variation in this measure. These inter‐correlations among summary statistics for individual determinands were unsurprising as the same data were summarised in a variety of ways, with several measures clearly related to one another (e.g., maximum, Q_95_ and Q_90_ all represent peaks). The pattern of correlation among summary statistics within determinands (Figure S1) reflected how normal the data were and how the measures stabilised over time, whereas the pattern of correlation among determinands (Table 2; Figure S2) is more likely to reflect associations in delivery and transformation of matter. Unsurprisingly, associated determinands (e.g., TP and SRP, TN and NO_3_‐N, velocity and discharge) were strongly correlated, whereas unrelated determinands were not (Table 2).

Likewsie, there was considerable correlation among the indices derived from the macroinvertebrate data (Figure 2; Table S4), with correlation most apparent among the stressor‐specific indices (ASPT_WHPT_, TRPI, PSI, ePSI, CoFSI, LIFE and SPEAR_pesticide_). The most strongly correlated indices were PSI and LIFE (across all sites *R* = 0.942). Correlations which included TRPI were not as strong as with other pairs of indices (Table S4) as TRPI cannot be calculated on macroinvertebrate samples collected in summer, thus reducing statistical power. For several pairs of indices, site had no significant influence on the relationship, which was the same both among sites and within sites (Table S4).

**FIGURE 3:**
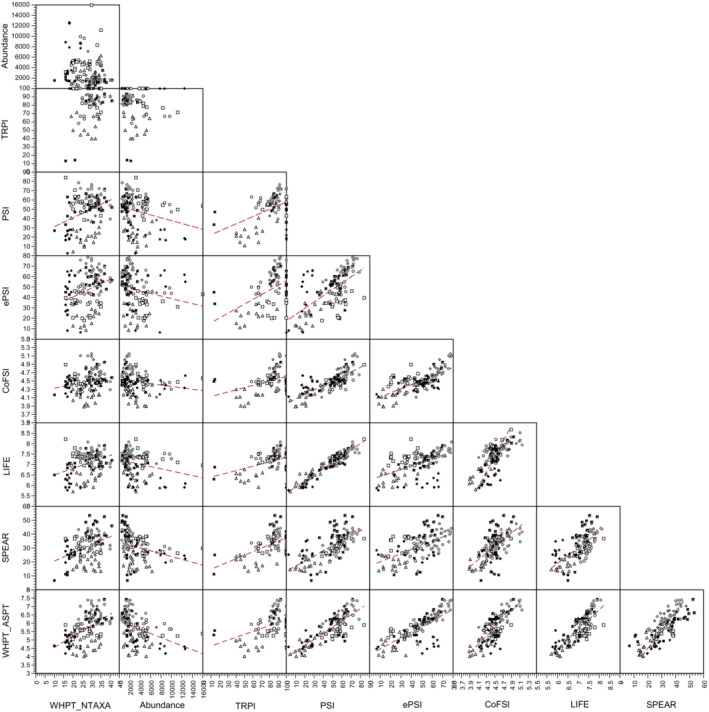
Relationships among macroinvertebrate based indices, with sites represented by different symbols. In all cases, the different indices were calculated using data derived from the same individual samples. For correlation coefficients and influence of site on relationships see Table S4.

When the biological indices were compared with single hydrochemical variables, summary statistics describing troughs in DO over relatively short time periods (1–10 days) best explained variation in all biotic indices except NTAXA_WHPT_, which was inversely correlated with mean TN over 20 days, and log_10_ Abundance, which was inversely correlated with peaks in velocity (Q_95_) over 60 days (Table 3). When site differences were included in the model, most of the variation was explained by differences among sites. Temporal variation in biotic indices within sites was best explained by DO for CoFSI (Q_10_ over 1 day), and ASPT_WHPT_ and SPEAR_pesticide_ (Q_5_ over 10 days), TP for ePSI (range of central 50% of observations over 20 days), turbidity for LIFE (coefficient of variation over 10 days) and velocity for NTAXA_WHPT_ (range of central 80% of observations over 60 days) and log_10_ Abundance (Q_95_ over 60 days; Table 3). In the case of the stressor‐specific indices (SPEAR_pesticide_, CoFSI, ePSI, LIFE and ASPT_WHPT_), measures of troughs in DO, particularly Q_5_ over 10 days, explained almost as much within‐site temporal variation as the selected determinand (Figure 3). Only one of the stressor‐specific indices, ASPT_WHPT_ (i.e., low concentrations of DO) showed any substantial correlation with the pressure that they putatively represent (Table 3). Both weak relationships between putative stressors and associated indices (Table 3), and the lack of correlations between the putative stressors and low concentrations of DO (Table 2; Figure S2) would suggest that this finding is not a consequence of covariation between possible explanatory variables.

**TABLE 3:** Results of model selection indicating which hydrochemical determinands best explained variation in the biotic indices, which determinand was best when local influences were accounted for (by including site in the model) and the capability of the stressor that the indices putatively measure to explain variation in the index.

Index	Putative stressor	Single determinand			Including site			Putative stressor		
		Determinand	Summary statistic	*R* ^2^	Determinand	Summary statistic	*R* ^2^	Determinand	Summary statistic	*R* ^2^
Spear_pesticide_	Pesticides	DO	Q_5_ 10 days	0.696	DO	Q_5_ 10 days	0.767			
CoFSI	Fine sediment	DO	Q_10_ 1 day	0.553	DO	Min 1 day	0.727	Turbidity	Days 3 × Q_50_ 90 days	0.111
PSI	Fine sediment	DO	Q_5_ 10 days	0.554	TP	Range _50_ 20 days	0.726	Turbidity	Min 90 days	0.137
ePSI_mtl_	Fine sediment	DO	Q_5_ 1 days	0.546	Ammonium	Q10_10	0.8079	Turbidity	Min 90 days	0.121
TRPI	Total reactive phosphorus	DO	Coef Var^n^ 10 days	0.6223	Free Ammonia	Median_30	0.8595	SRP	Range 80_90	0.170
LIFE	Low flow	DO	Q_5_ 10 days	0.530	Turbidity	Coef Var^n^ 10 days	0.852	Discharge	Median 60 days	0.123
ASPT_WHPT_	Organic pollution	DO	Q_5_ 10 days	0.700	DO	Q_5_ 10 days	0.896	DO	Q_5_ 10 days	0.896
NTAXA_WHPT_	General degradation	TN	Mean 20 days	0.428	Velocity	Range_80_ 60 days	0.649			
log_10_ Abundance		Velocity	Q_95_ 60 days	0.370	Velocity	Q_95_ 60 days	0.604			

**FIGURE 4:**
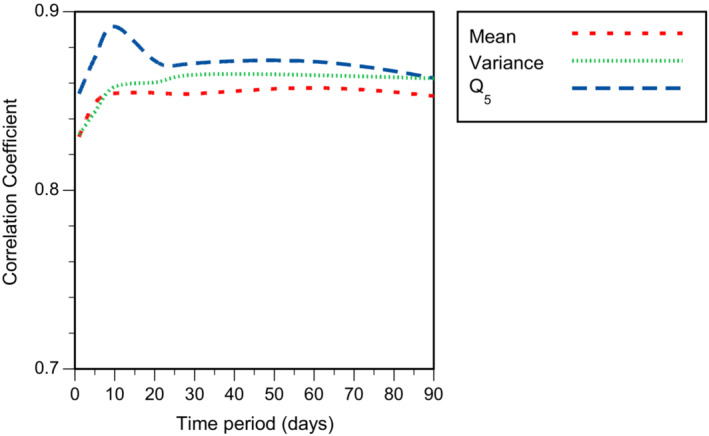
Influence of antecedent time period used to derive summary statistics (mean, variance, Q_5_) of dissolved oxygen (DO) conditions on the ability to describe variation in ASPT_WHPT_. For illustration of influence of length of observation period on summary statistics of DO, see Figure S3.

**FIGURE 5:**
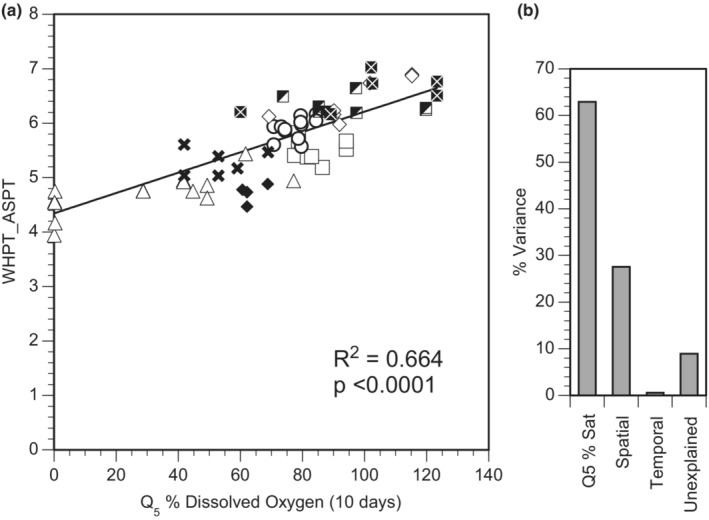
(a) Relationship between Q_5_ of dissolved oxygen (DO) conditions calculated over 10 days and ASPT_WHPT_ (different sites represented by different symbols) together with (b) the proportion of variance in ASPT_WHPT_ attributable to variation in Q_5_ of DO over 10 days, spatial differences among sites, temporal differences and unexplained.

**FIGURE 6:**
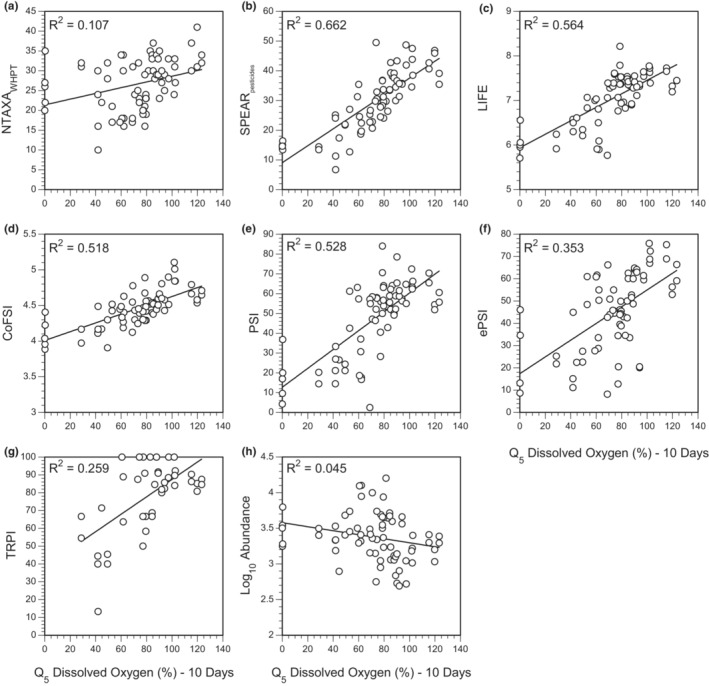
Relationships between (a) NTAXA_WHPT_, (b) Spear_pesticides_, (c) LIFE, (d) CoFSI, (e) PSI, (f) ePSI, (g) TRPI and (h) log_10_ Abundance, and Q_5_ of DO over 10 days.

When comparing across different antecedent time periods, it was apparent that Q_5_ of DO was best at describing variation in ASPT_WHPT_ when calculated over 10 days (Figure 4): although correlation increased initially with increasing time intervals, a similar peak in correlation was not apparent when DO was summarised as mean or variation. When calculated over 10 days, Q_5_ of DO explained 63% of the variation in ASPT_WHPT_, with 27% of the remaining variation attributable to spatial differences, <1% to temporal variation and 9% remaining unattributed (Figure 5). As well as describing the variation in ASPT_WHPT_, Q_5_ of DO described a large proportion of the variation in SPEAR_pesticide_, LIFE, CoFSI, PSI, ePSI and TRPI (Figure 6).

## 4 DISCUSSION

### 4.1 Can stressor‐specific indices diagnose multiple different stressors?

Although there has been a substantial effort to produce stressor‐specific indices that can be used to interpret ecological damage in terms that can be linked to management actions (Paisley et al., 2014; Turley et al., 2015, 2016), we have found that many of these stressor‐specific indices appear to be confounded by factors other than the pressure the index is supposed to assess. Despite ASPT_WHPT_ being the only stressor‐specific index that putatively responds to organic pollution, variation in all of the stressor‐specific indices used here (ASPT_WHPT_, TRPI, PSI, ePSI, CoFSI, LIFE and SPEAR_pesticide_) was most strongly correlated with low oxygen concentrations (Q_5_ of DO). Only NTAXA_WHPT_ (a measure of general degradation) and log_10_ Abundance did not appear to be correlated with DO saturation, with the latter negatively associated with peak flows (Q_95_ of velocity over 60 days). Whilst the approaches used here cannot determine cause‐and‐effect, Q_5_ of DO did not appear to be a surrogate for other water‐quality determinands more relevant to the indices in question. Furthermore, the seven stressor‐specific indices appear to be highly correlated with each other despite being putative measures of different stressors. This was particularly true of PSI (sedimentation) and LIFE (low flows), which varied in concert (*R* = 0.942) both within and between sites, a pattern that has been noted for the entire PSI family of indices (Murphy et al., 2015; Turley et al., 2014, 2016), leading to a conclusion that PSI and LIFE do not provide independent measures of the macroinvertebrate community.

As the biological data used here were derived from field samples collected from sites subject to diffuse pollution from agriculture, there are four mechanisms that could lead to correlation among biotic indices: common pressures, common pathways, common mode of action and common sensitivity.

At the catchment scale, agricultural activities can result in a number of different pressures acting simultaneously (Collins & McGonigle, 2008). For example, agricultural intensification can lead to increased fine sediment delivery from soil erosion, increased inorganic nutrient inputs from artificial fertilisers, and increased organic nutrient input from farmyard slurry, manure handling operations and outdoor livestock, as well as other potential effects (Collins et al., 2014). It is plausible that variation among sites reflected patterns in agricultural practice resulting in pressures acting in common. Likewise, many of the diffuse pollutants typically derived from agriculture follow common pathways of delivery to the river, usually driven by rainfall events (Kronvang et al., 1997; Lloyd et al., 2016b; Ng et al., 1993; Ockenden et al., 2017), which could accentuate the co‐occurrence of stressors. However, if either of these mechanisms (common pressures, common pathways) was the case, covariation in the hydrochemical determinands associated with the stressors that the indices putatively assess would be apparent. As none of the biotic indices were strongly correlated with the putative stressors they are supposed to reflect and, likewise, the putative stressors showed weak correlation with DO, it is unlikely that these two mechanisms were driving covariation in the stressor‐specific indices.

Another possible explanation for correlation among the indices, is that the stressors assessed have a common mode of action, where the proximal driver of change in the macroinvertebrate community is the same for the different indices. For example, low flows or an influx of organic matter (from sewage or agriculture) may cause depletion of DO in the benthic environment, whereby the low concentration of DO influences the macroinvertebrate community rather than low flows or organic pollution per se. Indeed, it is possible that the combined action of multiple stressors on the proximal driver could produce more pronounced effects on the biota than individual stressor alone (e.g., low flows exacerbate the effect of organic pollution on oxygen concentrations). But, again, if a common mode of action was driving covariation in the indices, the hydrochemical determinands associated with the stressors would be expected to co‐vary with the proximal driver, which appears to be low concentrations of DO. Such covariance between the putative drivers of the biotic indices and DO was not apparent.

The remaining, and mostly likely, explanation for correlation among the stressor‐specific indices is one of common sensitivity, whereby changes in the macroinvertebrate community caused by one stressor (in this case low oxygen concentrations) result in changes in the values returned by other indices, irrespective of variation in the underlying putative stressors. Additive effects of multiple stressors on communities have been noted before, where one stressor removes those taxa from the community that are tolerant to a second stressor (Vinebrooke et al., 2004), although synergistic or antagonistic effects also are possible. However, the correlation among stressor‐specific indices seen here is more likely to be a consequence of interpretation rather than the action of multiple stressors. As stressor‐specific indices attempt to interpret community composition in terms of individual stressors, any change in community could have consequences for returned values dependent on the relative scores of the taxa that change. The findings presented here indicate that low oxygen concentrations were strongly associated with change in invertebrate community composition, and it is likely that these changes affected the values returned for the stressor‐specific indices.

Support for common sensitivity being behind the correlation among stressor‐specific indices is provided by the two indices that used characteristics of the community other than species composition, NTAXA_WHPT_ and log_10_ Abundance, which are based on richness and abundance, respectively. These indices did not co‐vary with the other stressor‐specific indices, and were correlated with determinands other than oxygen, suggesting that variation in these indices was independent from the others. As the various stressor‐specific indices are different interpretations of the same information (species composition) they are vulnerable to being confounded by common sensitivity. However, it may be possible to extract different lines of useful information from biotic data where they are based on independent aspects of variation in the community.

Such interactions among indices caused by common sensitivity present a considerable challenge for interpretation of the causes of degradation: with so many options for mitigation of sources and pathways of transfer of pollutants, it is vital that we have a sound understanding of the potential interactions between stressors. In particular, we must understand how interacting stressors may confound interpretation of the causes of degradation, leading to costly yet ineffective mitigation of sources that are not the sole or dominant cause of stress in the system. In the data presented here, we have shown that low saturation of oxygen (particularly Q_5_ over 10 days) appears to be most strongly associated with change in macroinvertebrate community composition, which, in turn, leads to variation in the returned values of stressor‐specific indices. Given the covariation in stressor‐specific indices identified here, we suggest that additional independent evidence should be used to corroborate any conclusions regarding the causes of degradation based on any of the stressor‐specific indices tested other than ASPT_WHPT_. Such evidence may be from surveys of hydrochemistry, land use and management, independent measures of biological response or models that include any of the above.

### 4.2 Do episodic events cause lasting changes in biotic index values?

Another aspect of biotic indices, which has received less attention than relationships with stressors, is the temporal scale of the biological response. Biological communities are dynamic. The resilience of communities to episodic events both influences the period that is reflected in indices and has important consequences for the response to changes in pressures. The principle of space‐for‐time can lead to the presumption that change in the pressure at a site will result in a response similar to that seen in differences among sites, which may not be true. The rate and extent of response of biological communities to change in pressures will depend on the resilience of the community, and there are likely to be lags in response where resilience is dependent upon larger scale influences, such as rate of colonisation.

The analysis of the influence of antecedent conditions on biota undertaken here attempts to capture the temporal scales over which conditions relevant to the sampled community occur. Although it is possible that the most recent events have most influence on the community, shorter antecedent time periods may exclude prior influential events, thus introducing noise into the relationship. Likewise, if there is a time lag in the biological response, shorter time periods will not include events that precipitated the change. At some point, largely determined by the rate of change in the community and the frequency of influential events, an optimal antecedent period will be found which is best at capturing those events that have the most influence.

In terms of the trait reflected by ASPT_WHPT_, the invertebrate communities sampled here were, to an extent, capable of rapid recovery. A peak in correlation between ASPT_WHPT_ and DO was obtained when an antecedent period of 10 days was used to calculate Q_5_, indicating that earlier episodes of low oxygen saturation had less influence on the macroinvertebrate community sampled. Whilst low DO events are likely to cause some lasting impact, the community had recovered to some extent from low DO episodes that occurred more than 10 days before sampling. Such rapid recovery was likely to have been facilitated by rapid, probably small scale processes, such as behavioural avoidance of adverse conditions (Edwards et al., 1991) or recolonisation from local, well‐connected populations. As a consequence of the way ASPT_WHPT_ is calculated (average score per taxon) the occurrence of sensitive taxa can have a substantial influence, potentially leading to a rapid response. On the other hand, log_10_ Abundance of macroinvertebrates appeared to be negatively influenced by peak flows (Q_95_ of velocity calculated over 60 days). The disturbance associated with peak flows is likely to have reduced the abundance of macroinvertebrates (Dunbar et al., 2010; Gjerløv et al., 2003), and it is plausible that recovery of abundance is dependent on slower processes (population growth/recolonisation) than those affecting ASPT_WHPT_.

Nevertheless, periods of low DO appeared to have a substantial influence on the macroinvertebrate community: a well‐known phenomenon (Hynes, 1960). The majority of variation (63%) in ASPT_WHPT_ was attributable to Q_5_ of DO over 10 days, with 27% attributable to differences among sites, and >1% to temporal differences, thus largely supporting the assumption that differences in ASPT_WHPT_ among sites are comparable to change within sites. Likewise, the other stressor‐specific indices were correlated with low DO, although this is likely to be a consequence of how change in the macroinvertebrate community influenced the returned values of indices. Whilst the results of the work here support the principle of space‐for‐time for ASPT_WHPT_, they also raise a concern about interpretation of stressor‐specific indices. More effort should be made to establish the interdependence of biotic indices before they are adopted by management. Furthermore, given the implications of incorrect assignment of causes of ecological degradation (unjustified burdens being placed on catchment stakeholders), we repeat that these indices should not be used in isolation to diagnose the causes of degradation, and that additional evidence should be used to corroborate any conclusions drawn from them. The use of such stressor‐specific indices alone risks the mistargeting of management strategies if the putative stressor‐index approach is taken to be more reliable than the results herein suggest.

## 5 CONCLUSIONS

The returned values of the stressor‐specific indices PSI, ePSI, CoFSI, LIFE, TRPI and SPEAR_pesticide_ appear to be confounded by changes in the macroinvertebrate community driven by stressors other than those they putatively assess, particularly periods of low oxygen saturation. ASPT_WHPT_ was most strongly correlated with Q_5_ of DO over the preceding 10 days, which explained the majority (67%) of variation both among and within sites, indicating that earlier episodes of low oxygen saturation had less influence on the macroinvertebrate community sampled. Whilst periods of low DO are likely to cause some lasting impact, the community had recovered to some extent from earlier low DO episodes. Total abundance appeared slower to respond, with Q_95_ of velocity calculated over 60 days being the best at explaining variation, suggesting that recovery in macroinvertebrate numbers is slower than recovery of composition. To a large extent, the principle of space‐for‐time was supported, but we suggest caution as the processes that govern biological response times may vary dependent on the nature and extent of impacts. We also stress caution regarding the interpretation of stressor‐specific indices. Stressor‐specific indices (other than ASPT_WHPT_ and its forerunner ASPT_BMWP_) should not be used in isolation to diagnose the causes of degradation of sites and require additional evidence to corroborate any conclusions regarding the causes of degradation drawn from them.

## AUTHOR CONTRIBUTIONS

JIJ, CEML, JFM, JEF, AA, PJJ, ALC: Conceptualisation. ALC, CEML, JIJ, PJJ, JEF, JFM: Developing methods. JIJ, AA, JFM, CEML: Preparation of figures and tables. JIJ, CEML, JFM, AA, CPD, AH, JLP, PJJ, JEF, MWS, CR, ALC: Conducting the research, data interpretation, writing.

## CONFLICT OF INTEREST STATEMENT

The authors declare no conflicts of interest.

## Supporting information


Figure S1.

Figure S2.

Figure S3.

Table S1.

## Data Availability

Data used in this study are available from http://www.environmentdata.org/dtc‐archive‐project.
