# Supplementary material for: What do macroinvertebrate indices measure? Stressor‐specific stream macroinvertebrate indices can be confounded by other stressors
Source: Freshw Biol. 2023 May 17;68(8):1330–45. doi: 10.1111/fwb.14106 (PMC10952762; doi:10.1111/fwb.14106)
Supplement: Supplementary file 1 — Figure S1. [file FWB-68-1330-s004.pdf]

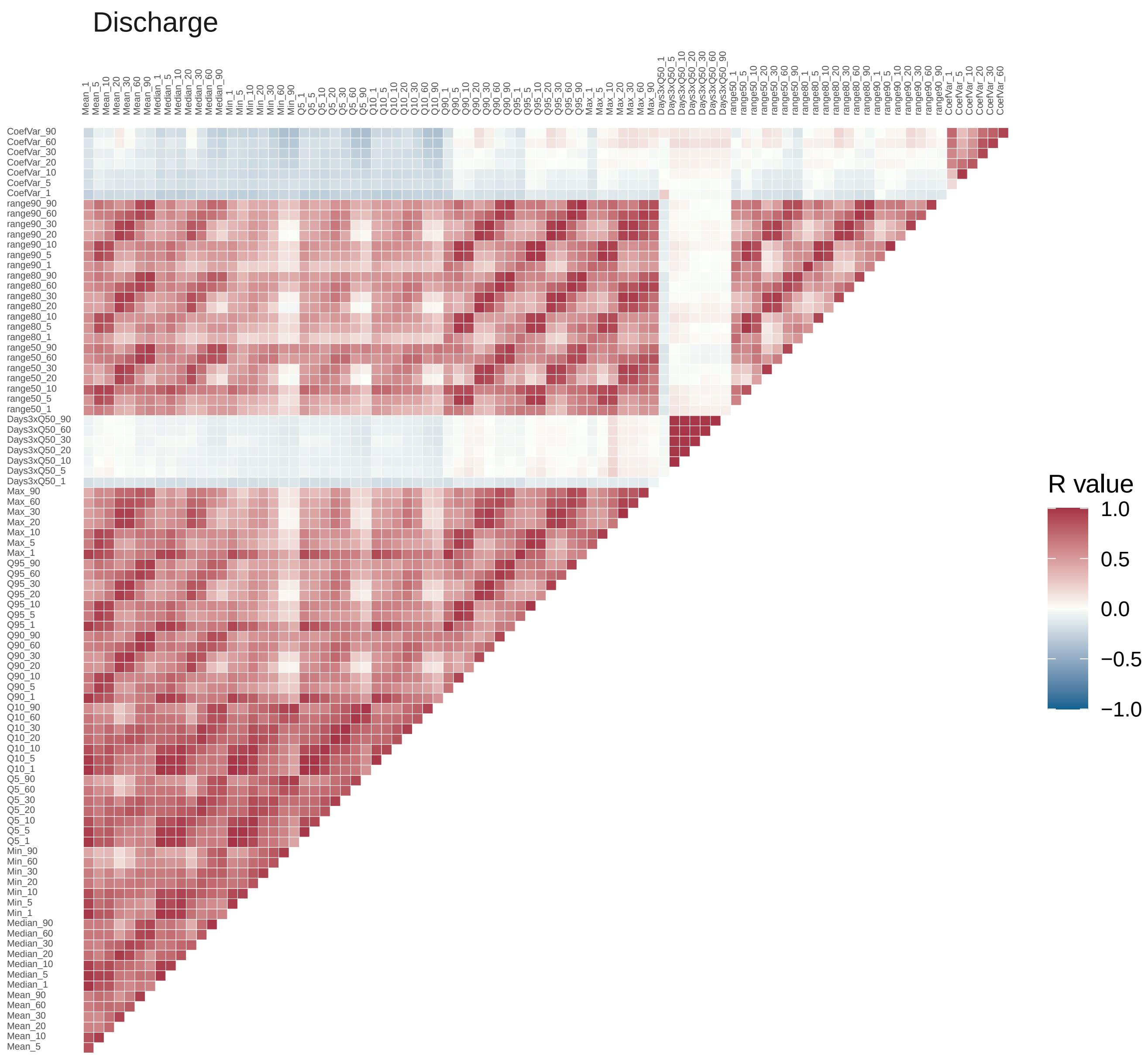
Discharge
R value
1.0
0.5
0.0
−0.5
−1.0
CoefVar_90
CoefVar_60
CoefVar_30
CoefVar_20
CoefVar_10
CoefVar_5
CoefVar_1
range90_90
range90_60
range90_30
range90_20
range90_10
range90_5
range90_1
range80_90
range80_60
range80_30
range80_20
range80_10
range80_5
range80_1
range50_90
range50_60
range50_30
range50_20
range50_10
range50_5
range50_1
Days3xQ50_90
Days3xQ50_60
Days3xQ50_30
Days3xQ50_20
Days3xQ50_10
Days3xQ50_5
Days3xQ50_1
Max_90
Max_60
Max_30
Max_20
Max_10
Max_5
Max_1
Q95_90
Q95_60
Q95_30
Q95_20
Q95_10
Q95_5
Q95_1
Q90_90
Q90_60
Q90_30
Q90_20
Q90_10
Q90_5
Q90_1
Q10_90
Q10_60
Q10_30
Q10_20
Q10_10
Q10_5
Q10_1
Q5_90
Q5_60
Q5_30
Q5_20
Q5_10
Q5_5
Q5_1
Min_90
Min_60
Min_30
Min_20
Min_10
Min_5
Min_1
Median_90
Median_60
Median_30
Median_20
Median_10
Median_5
Median_1
Mean_90
Mean_60
Mean_30
Mean_20
Mean_10
Mean_5
Mean_1

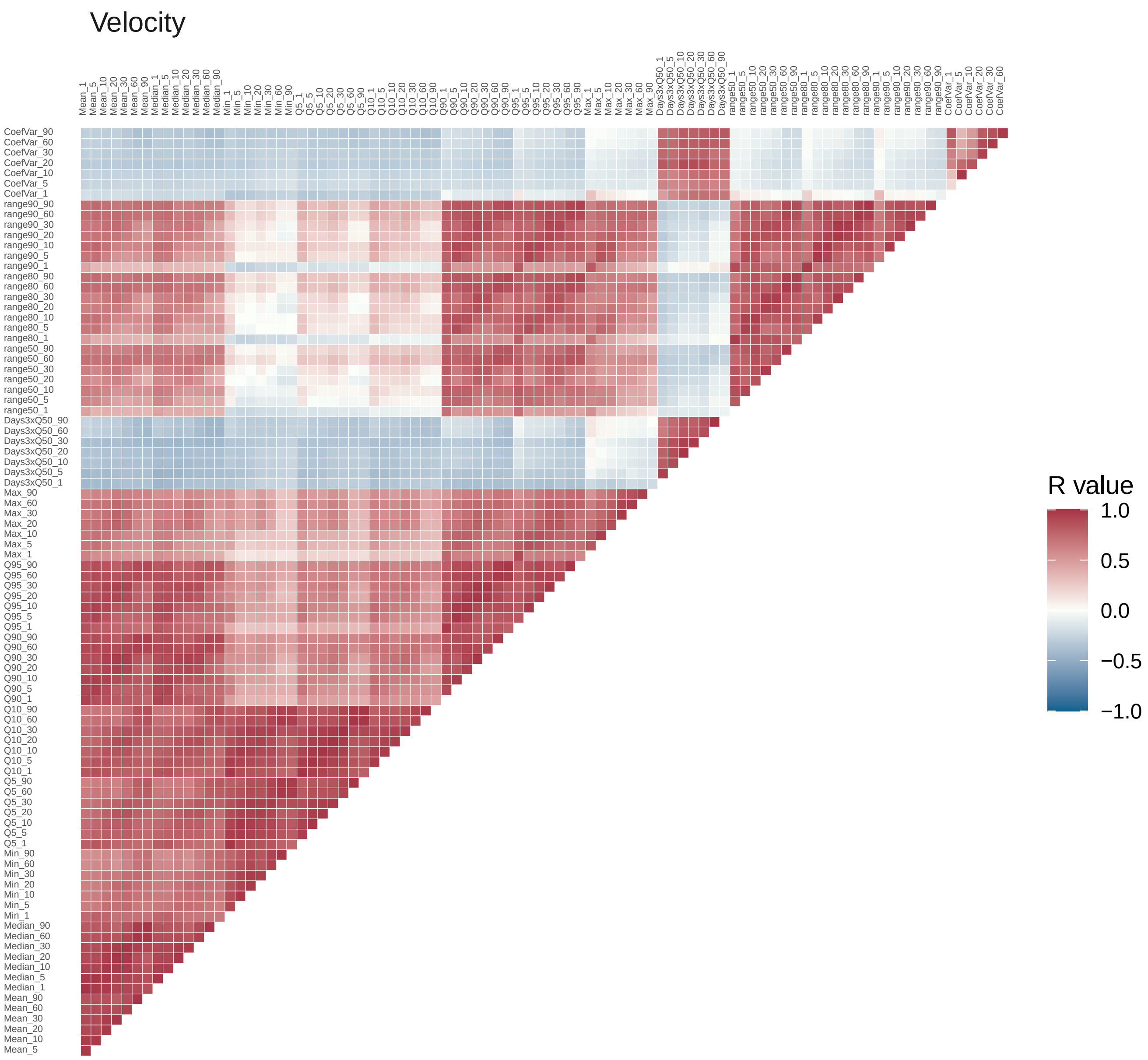
Velocity
R value
1.0
0.5
0.0
−0.5
−1.0

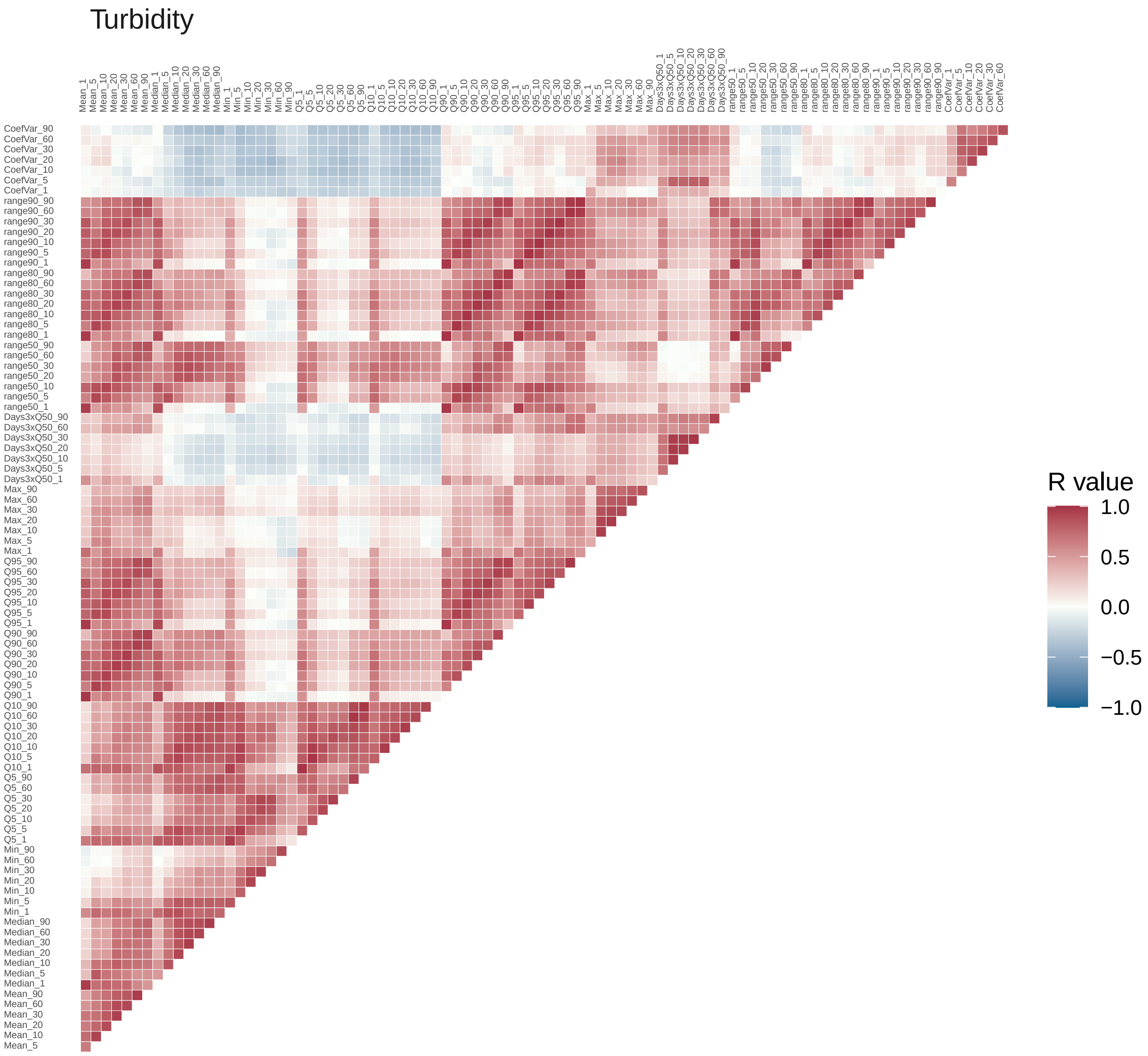
Turbidity
R value
1.0
0.5
0.0
−0.5
−1.0
Mean_1
Mean_5
Mean_10
Mean_20
Mean_30
Mean_60
Mean_90
Median_1
Median_5
Median_10
Median_20
Median_30
Median_60
Median_90
Min_1
Min_5
Min_10
Min_20
Min_30
Min_60
Min_90
Q5_1
Q5_5
Q5_10
Q5_20
Q5_30
Q5_60
Q5_90
Q10_1
Q10_5
Q10_10
Q10_20
Q10_30
Q10_60
Q10_90
Q90_1
Q90_5
Q90_10
Q90_20
Q90_30
Q90_60
Q90_90
Q95_1
Q95_5
Q95_10
Q95_20
Q95_30
Q95_60
Q95_90
Max_1
Max_5
Max_10
Max_20
Max_30
Max_60
Max_90
Days3xQ50_1
Days3xQ50_5
Days3xQ50_10
Days3xQ50_20
Days3xQ50_30
Days3xQ50_60
Days3xQ50_90
range50_1
range50_5
range50_10
range50_20
range50_30
range50_60
range50_90
range80_1
range80_5
range80_10
range80_20
range80_30
range80_60
range80_90
range90_1
range90_5
range90_10
range90_20
range90_30
range90_60
range90_90
CoefVar_1
CoefVar_5
CoefVar_10
CoefVar_20
CoefVar_30
CoefVar_60
CoefVar_90

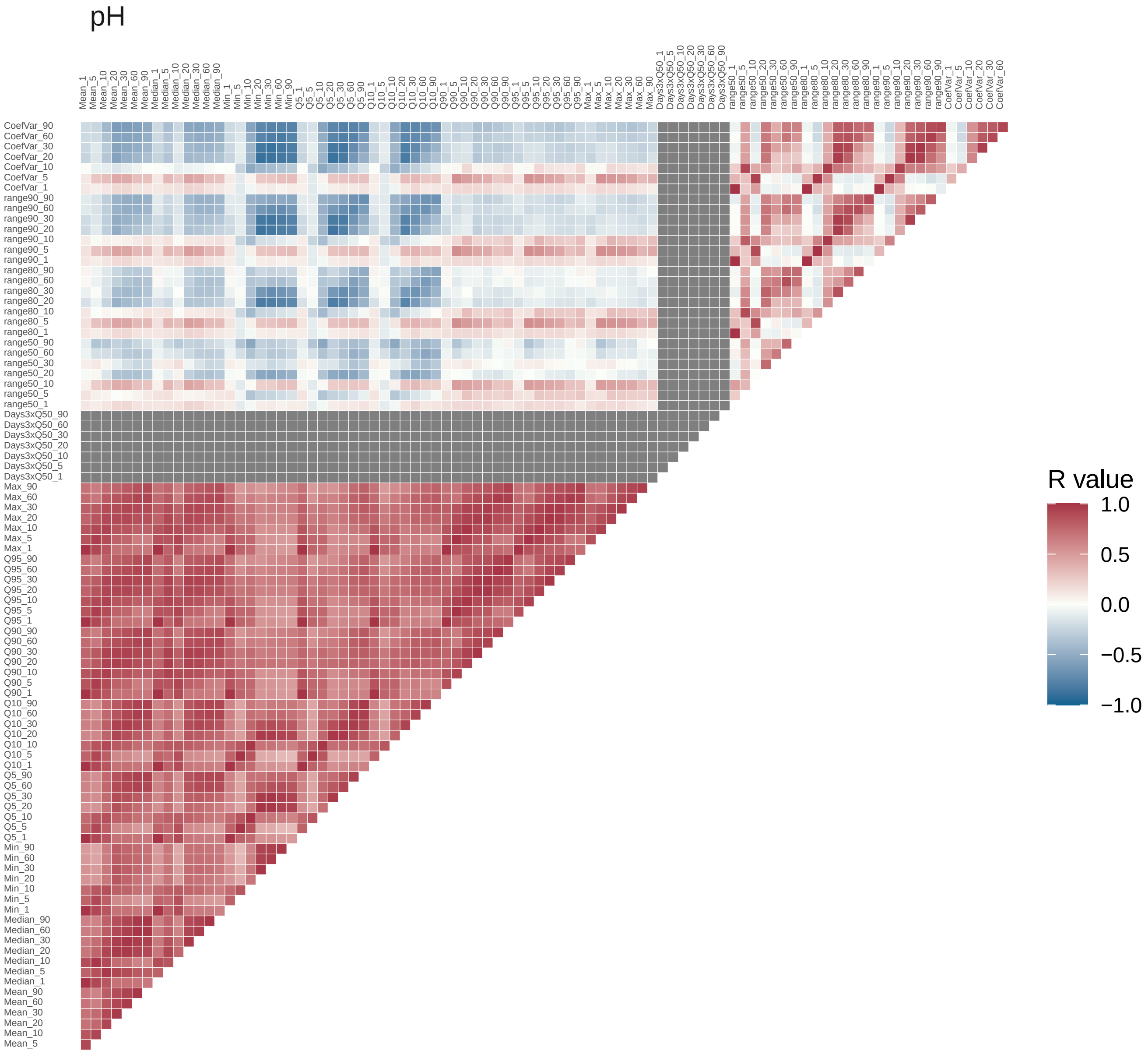
pH
R value
1.0
0.5
0.0
−0.5
−1.0

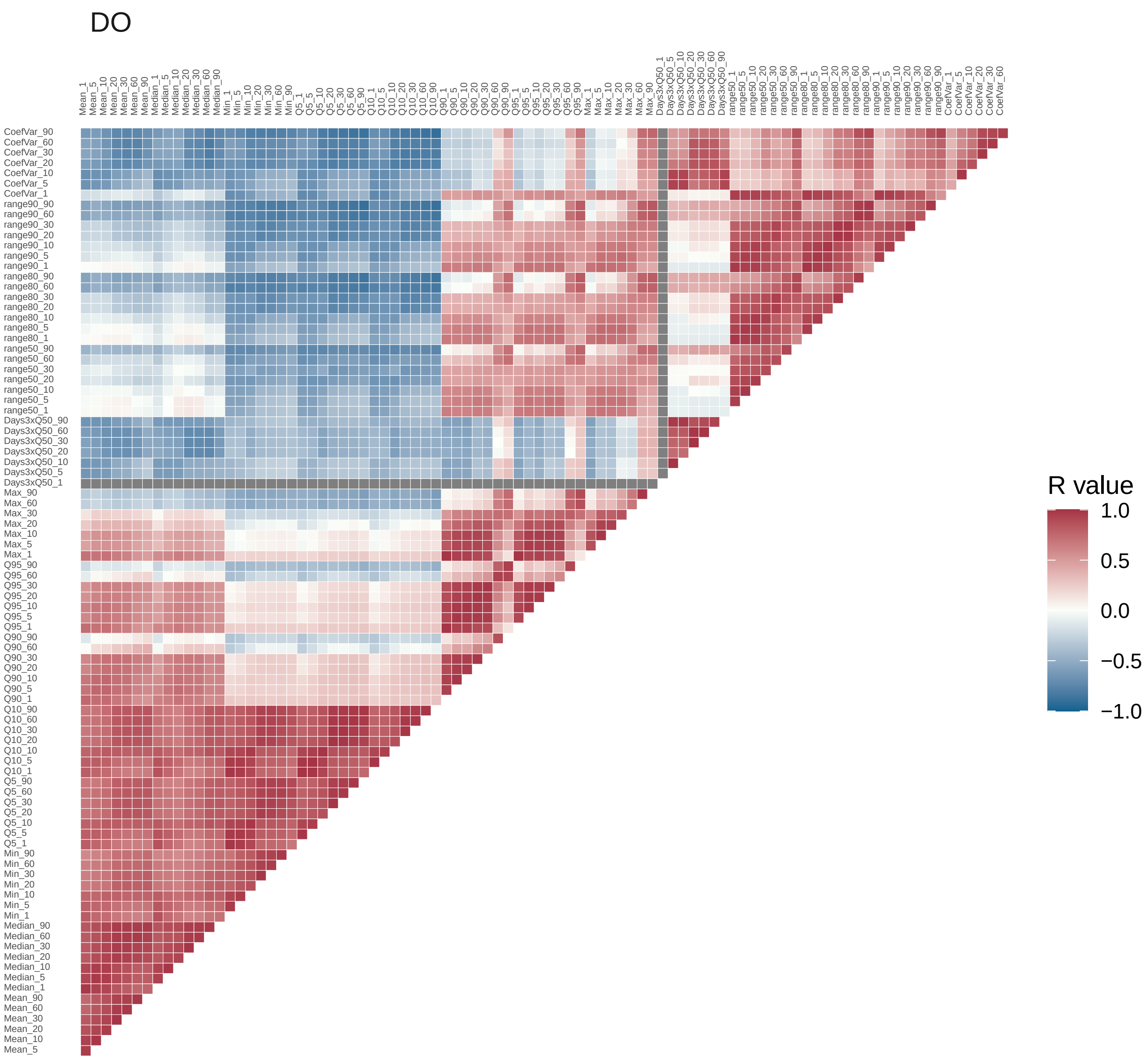
DO
Mean_1
Mean_5
Mean_10
Mean_20
Mean_30
Mean_60
Mean_90
Median_1
Median_5
Median_10
Median_20
Median_30
Median_60
Median_90
Min_1
Min_5
Min_10
Min_20
Min_30
Min_60
Min_90
Q5_1
Q5_5
Q5_10
Q5_20
Q5_30
Q5_60
Q5_90
Q10_1
Q10_5
Q10_10
Q10_20
Q10_30
Q10_60
Q10_90
Q90_1
Q90_5
Q90_10
Q90_20
Q90_30
Q90_60
Q90_90
Q95_1
Q95_5
Q95_10
Q95_20
Q95_30
Q95_60
Q95_90
Max_1
Max_5
Max_10
Max_20
Max_30
Max_60
Max_90
Days3xQ50_1
Days3xQ50_5
Days3xQ50_10
Days3xQ50_20
Days3xQ50_30
Days3xQ50_60
Days3xQ50_90
range50_1
range50_5
range50_10
range50_20
range50_30
range50_60
range50_90
range80_1
range80_5
range80_10
range80_20
range80_30
range80_60
range80_90
range90_1
range90_5
range90_10
range90_20
range90_30
range90_60
range90_90
CoefVar_1
CoefVar_5
CoefVar_10
CoefVar_20
CoefVar_30
CoefVar_60
CoefVar_90
R value
1.0
0.5
0.0
−0.5
−1.0

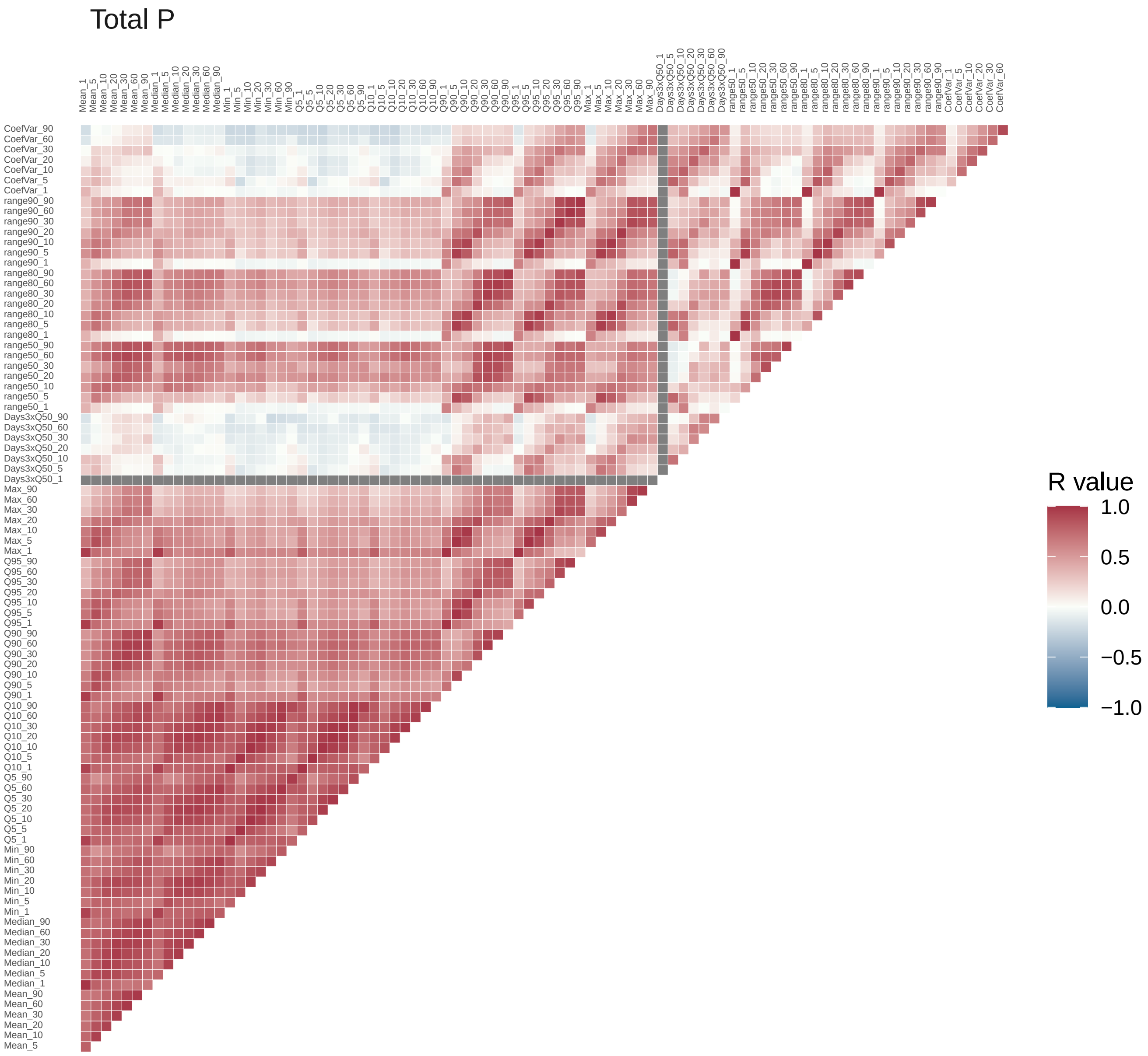

Total P
R value
1.0
0.5
0.0
−0.5
−1.0

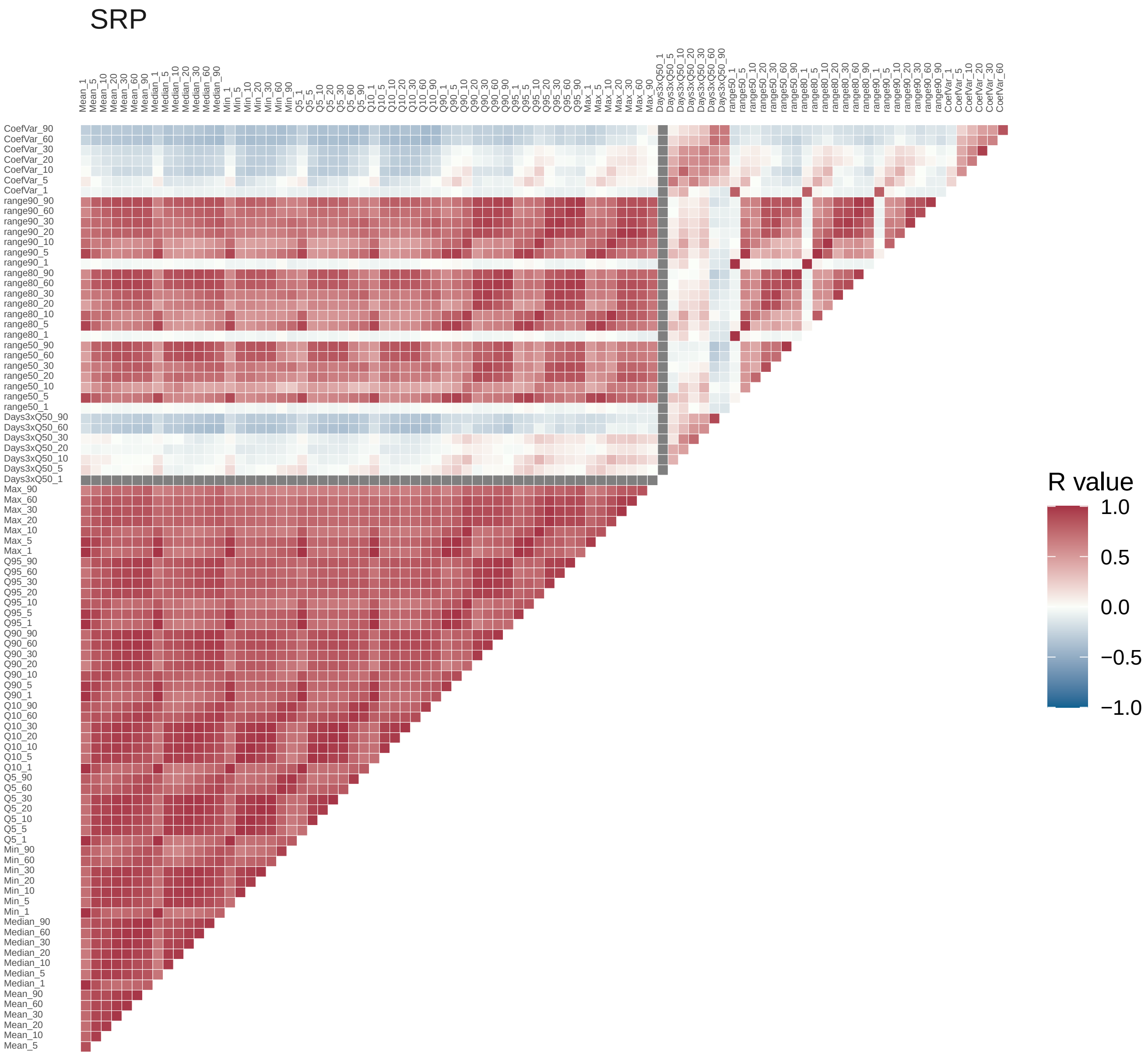
SRP
Mean_1
Mean_5
Mean_10
Mean_20
Mean_30
Mean_60
Mean_90
Median_1
Median_5
Median_10
Median_20
Median_30
Median_60
Median_90
Min_1
Min_5
Min_10
Min_20
Min_30
Min_60
Min_90
Q5_1
Q5_5
Q5_10
Q5_20
Q5_30
Q5_60
Q5_90
Q10_1
Q10_5
Q10_10
Q10_20
Q10_30
Q10_60
Q10_90
Q90_1
Q90_5
Q90_10
Q90_20
Q90_30
Q90_60
Q90_90
Q95_1
Q95_5
Q95_10
Q95_20
Q95_30
Q95_60
Q95_90
Max_1
Max_5
Max_10
Max_20
Max_30
Max_60
Max_90
Days3xQ50_1
Days3xQ50_5
Days3xQ50_10
Days3xQ50_20
Days3xQ50_30
Days3xQ50_60
Days3xQ50_90
range50_1
range50_5
range50_10
range50_20
range50_30
range50_60
range50_90
range80_1
range80_5
range80_10
range80_20
range80_30
range80_60
range80_90
range90_1
range90_5
range90_10
range90_20
range90_30
range90_60
range90_90
CoefVar_1
CoefVar_5
CoefVar_10
CoefVar_20
CoefVar_30
CoefVar_60
CoefVar_90
CoefVar_60
CoefVar_30
CoefVar_20
CoefVar_10
CoefVar_5
CoefVar_1
range90_90
range90_60
range90_30
range90_20
range90_10
range90_5
range90_1
range80_90
range80_60
range80_30
range80_20
range80_10
range80_5
range80_1
range50_90
range50_60
range50_30
range50_20
range50_10
range50_5
range50_1
Days3xQ50_90
Days3xQ50_60
Days3xQ50_30
Days3xQ50_20
Days3xQ50_10
Days3xQ50_5
Days3xQ50_1
Max_90
Max_60
Max_30
Max_20
Max_10
Max_5
Max_1
Q95_90
Q95_60
Q95_30
Q95_20
Q95_10
Q95_5
Q95_1
Q90_90
Q90_60
Q90_30
Q90_20
Q90_10
Q90_5
Q90_1
Q10_90
Q10_60
Q10_30
Q10_20
Q10_10
Q10_5
Q10_1
Q5_90
Q5_60
Q5_30
Q5_20
Q5_10
Q5_5
Q5_1
Min_90
Min_60
Min_30
Min_20
Min_10
Min_5
Min_1
Median_90
Median_60
Median_30
Median_20
Median_10
Median_5
Median_1
Mean_90
Mean_60
Mean_30
Mean_20
Mean_10
Mean_5
R value
1.0
0.5
0.0
−0.5
−1.0

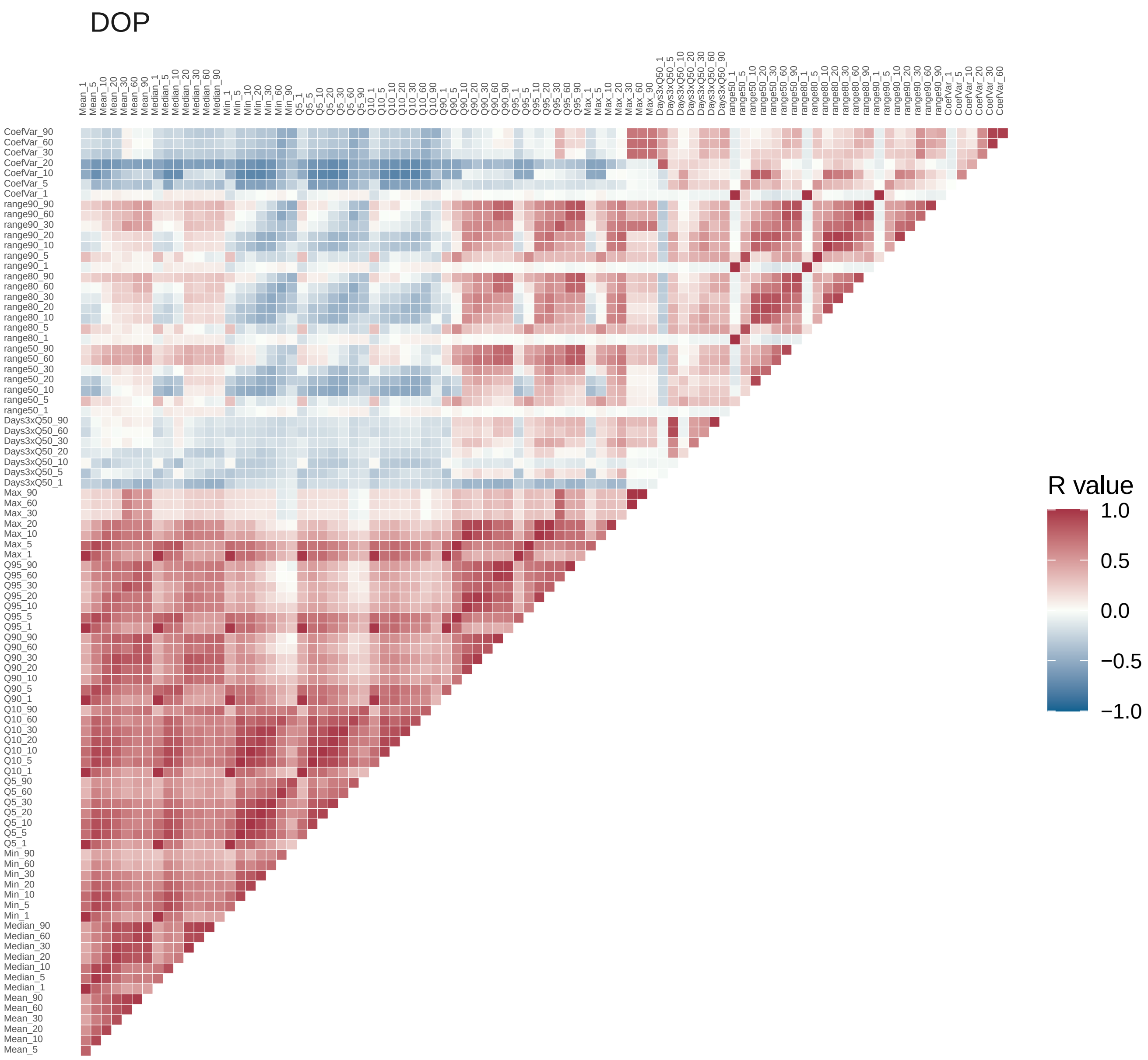
DOP
R value
1.0
0.5
0.0
−0.5
−1.0

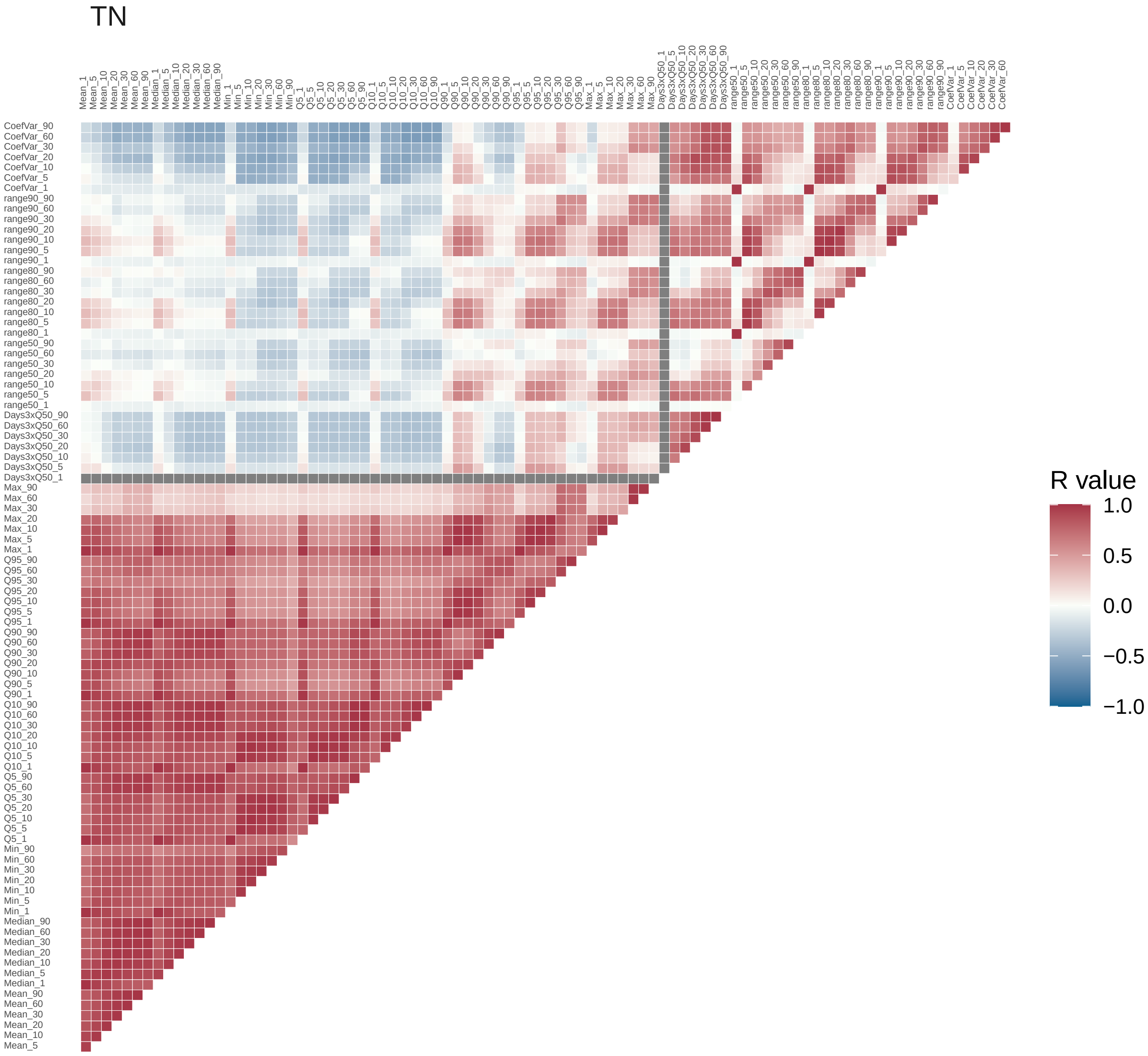
TN
R value
1.0
0.5
0.0
−0.5
−1.0

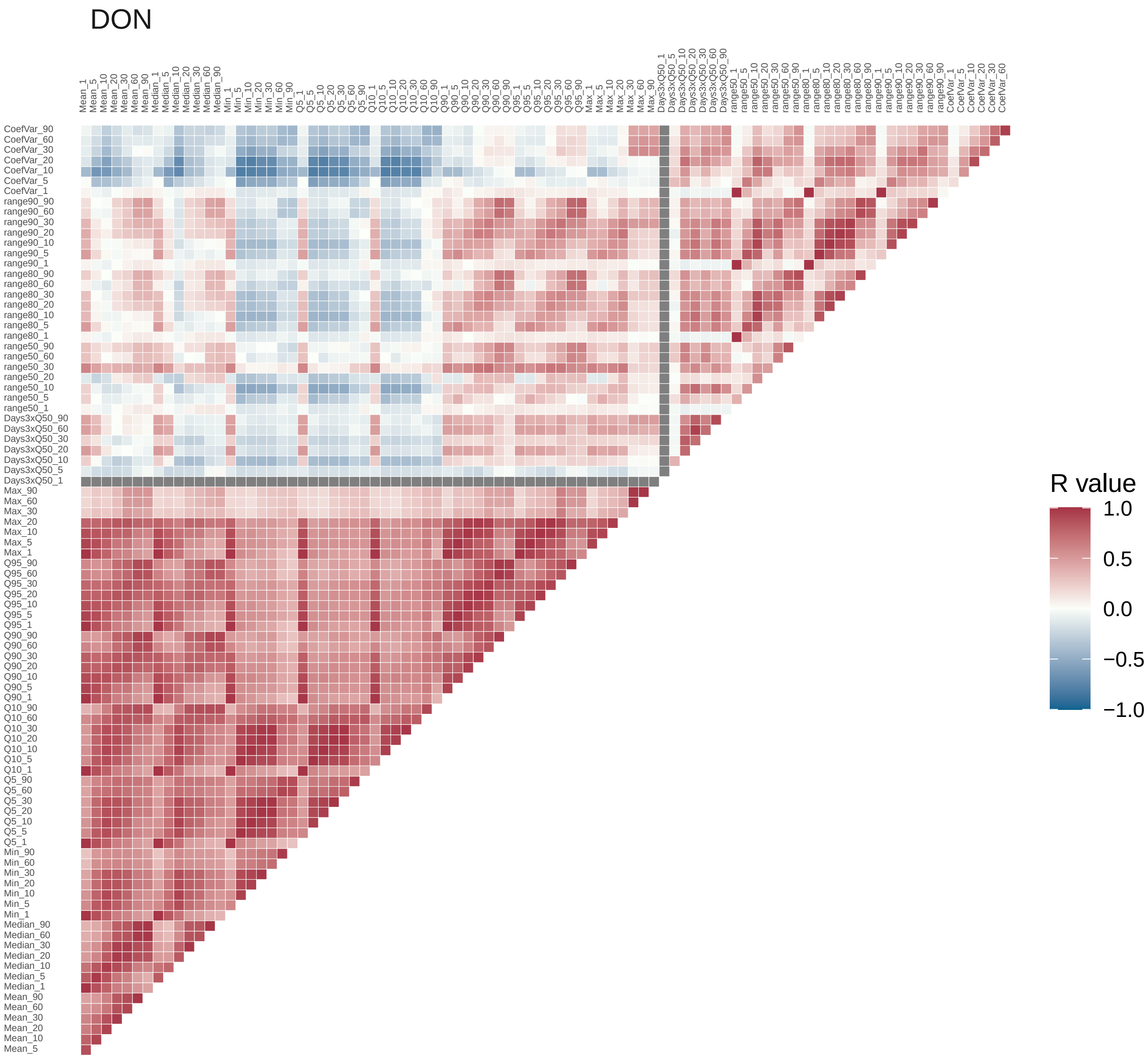
DON
Mean_1
Mean_5
Mean_10
Mean_20
Mean_30
Mean_60
Mean_90
Median_1
Median_5
Median_10
Median_20
Median_30
Median_60
Median_90
Min_1
Min_5
Min_10
Min_20
Min_30
Min_60
Min_90
Q5_1
Q5_5
Q5_10
Q5_20
Q5_30
Q5_60
Q5_90
Q10_1
Q10_5
Q10_10
Q10_20
Q10_30
Q10_60
Q10_90
Q90_1
Q90_5
Q90_10
Q90_20
Q90_30
Q90_60
Q90_90
Q95_1
Q95_5
Q95_10
Q95_20
Q95_30
Q95_60
Q95_90
Max_1
Max_5
Max_10
Max_20
Max_30
Max_60
Max_90
Days3xQ50_1
Days3xQ50_5
Days3xQ50_10
Days3xQ50_20
Days3xQ50_30
Days3xQ50_60
Days3xQ50_90
range50_1
range50_5
range50_10
range50_20
range50_30
range50_60
range50_90
range80_1
range80_5
range80_10
range80_20
range80_30
range80_60
range80_90
range90_1
range90_5
range90_10
range90_20
range90_30
range90_60
range90_90
CoefVar_1
CoefVar_5
CoefVar_10
CoefVar_20
CoefVar_30
CoefVar_60
CoefVar_90
R value
1.0
0.5
0.0
−0.5
−1.0

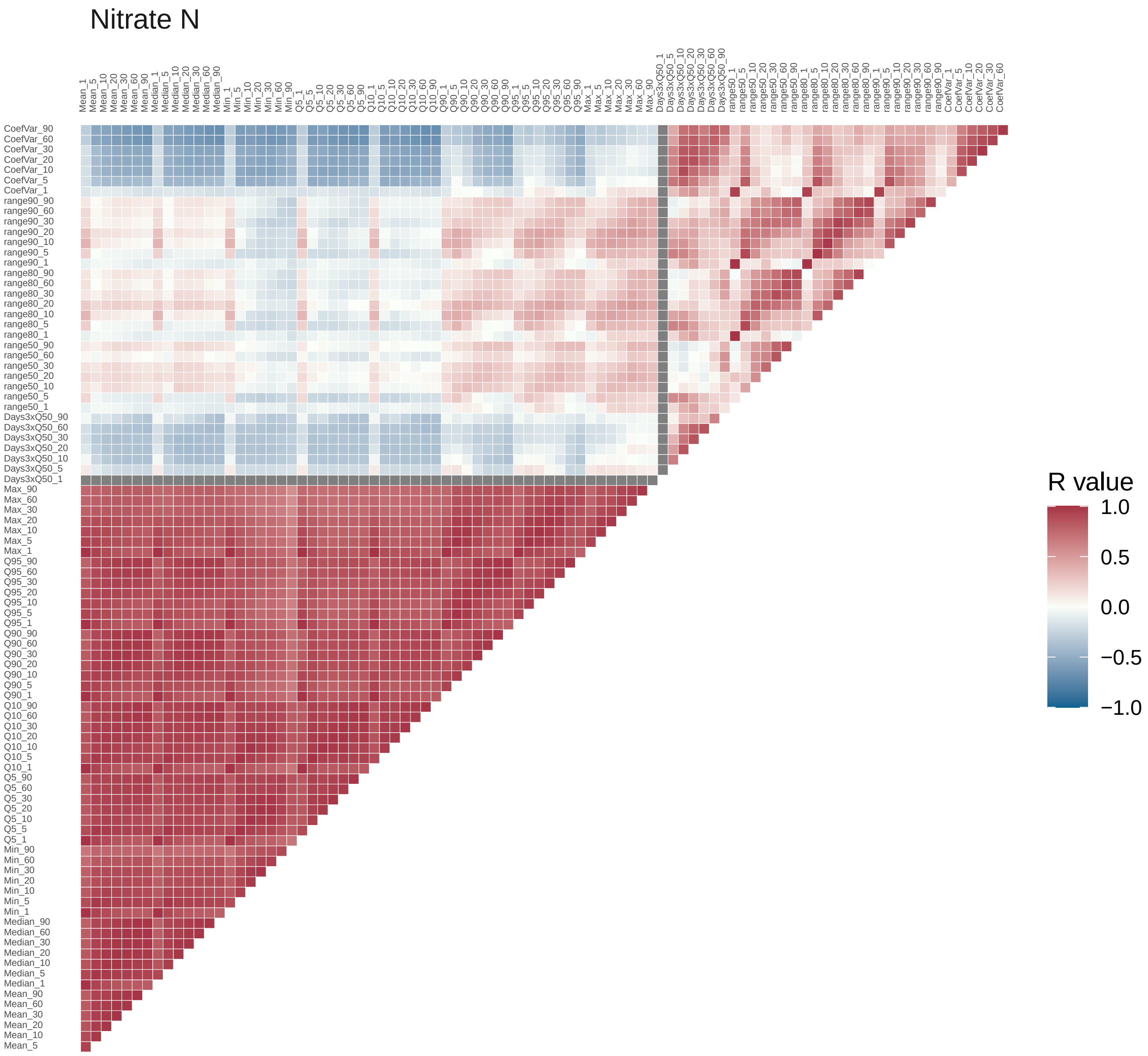
Nitrate N
R value
1.0
0.5
0.0
−0.5
−1.0

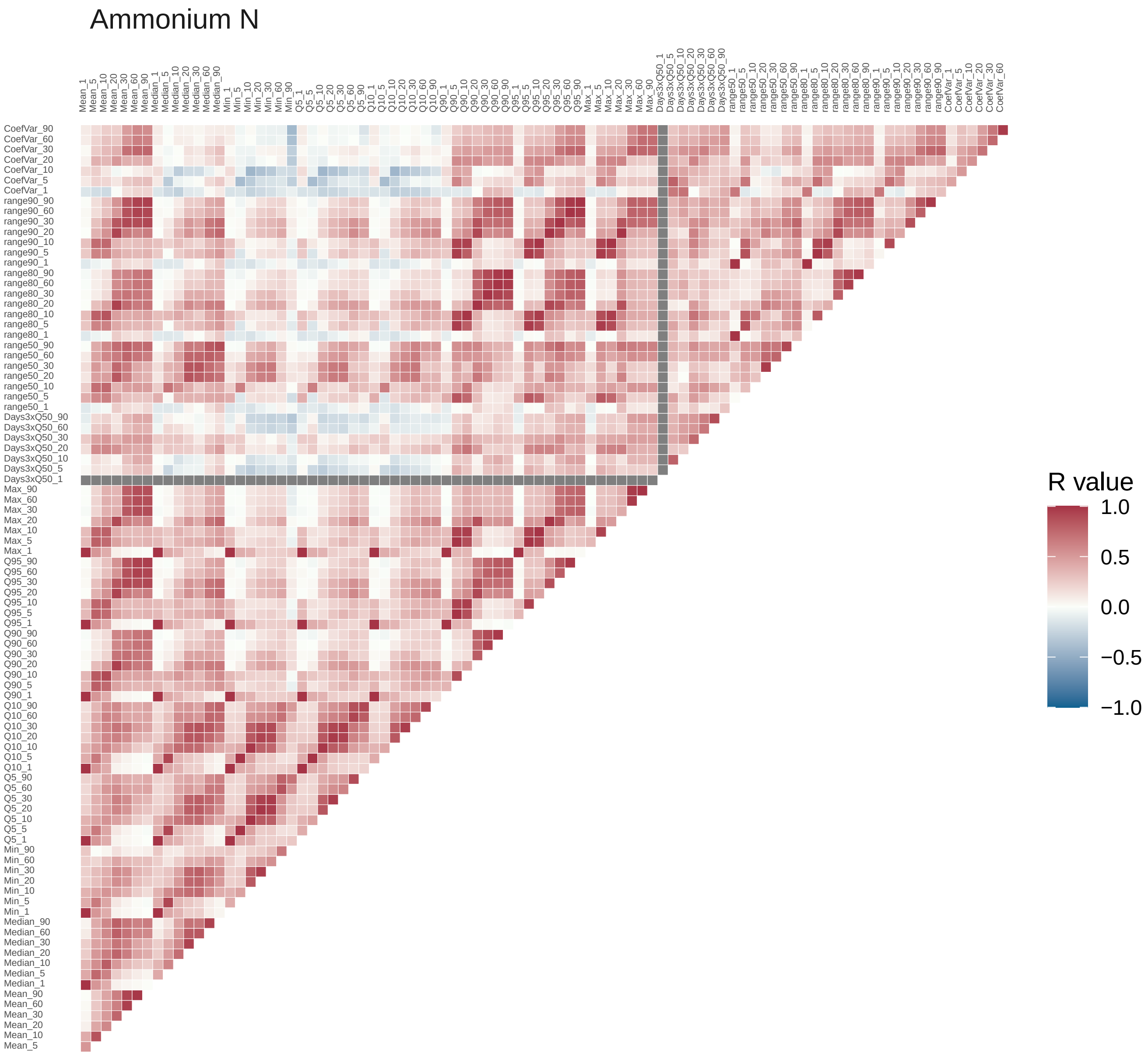

Ammonium N
R value
1.0
0.5
0.0
−0.5
−1.0

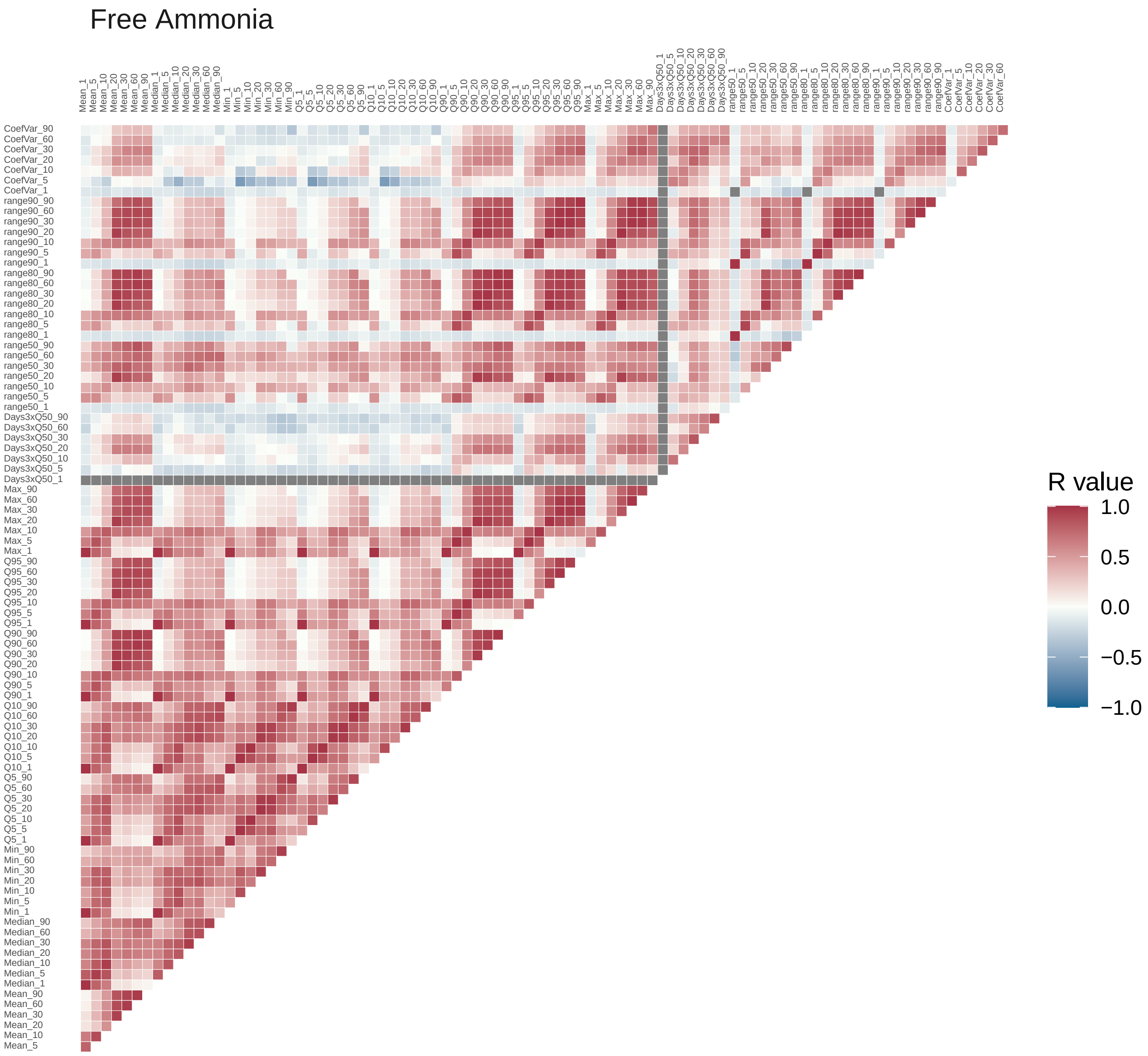
Free Ammonia
Mean_1 Mean_5 Mean_10 Mean_20 Mean_30 Mean_60 Mean_90 Median_1 Median_5 Median_10 Median_20 Median_30 Median_60 Median_90 Min_1 Min_5 Min_10 Min_20 Min_30 Min_60 Min_90 Q5_1 Q5_5 Q5_10 Q5_20 Q5_30 Q5_60 Q5_90 Q10_1 Q10_5 Q10_10 Q10_20 Q10_30 Q10_60 Q10_90 Q90_1 Q90_5 Q90_10 Q90_20 Q90_30 Q90_60 Q90_90 Q95_1 Q95_5 Q95_10 Q95_20 Q95_30 Q95_60 Q95_90 Max_1 Max_5 Max_10 Max_20 Max_30 Max_60 Max_90 Days3xQ50_1 Days3xQ50_5 Days3xQ50_10 Days3xQ50_20 Days3xQ50_30 Days3xQ50_60 Days3xQ50_90 range50_1 range50_5 range50_10 range50_20 range50_30 range50_60 range50_90 range80_1 range80_5 range80_10 range80_20 range80_30 range80_60 range80_90 range90_1 range90_5 range90_10 range90_20 range90_30 range90_60 range90_90 CoefVar_1 CoefVar_5 CoefVar_10 CoefVar_20 CoefVar_30 CoefVar_60
CoefVar_90 CoefVar_60 CoefVar_30 CoefVar_20 CoefVar_10 CoefVar_5 CoefVar_1 range90_90 range90_60 range90_30 range90_20 range90_10 range90_5 range90_1 range80_90 range80_60 range80_30 range80_20 range80_10 range80_5 range80_1 range50_90 range50_60 range50_30 range50_20 range50_10 range50_5 range50_1 Days3xQ50_90 Days3xQ50_60 Days3xQ50_30 Days3xQ50_20 Days3xQ50_10 Days3xQ50_5 Days3xQ50_1 Max_90 Max_60 Max_30 Max_20 Max_10 Max_5 Max_1 Q95_90 Q95_60 Q95_30 Q95_20 Q95_10 Q95_5 Q95_1 Q90_90 Q90_60 Q90_30 Q90_20 Q90_10 Q90_5 Q90_1 Q10_90 Q10_60 Q10_30 Q10_20 Q10_10 Q10_5 Q10_1 Q5_90 Q5_60 Q5_30 Q5_20 Q5_10 Q5_5 Q5_1 Min_90 Min_60 Min_30 Min_20 Min_10 Min_5 Min_1 Median_90 Median_60 Median_30 Median_20 Median_10 Median_5 Median_1 Mean_90 Mean_60 Mean_30 Mean_20 Mean_10 Mean_5
R value
1.0
0.5
0.0
−0.5
−1.0

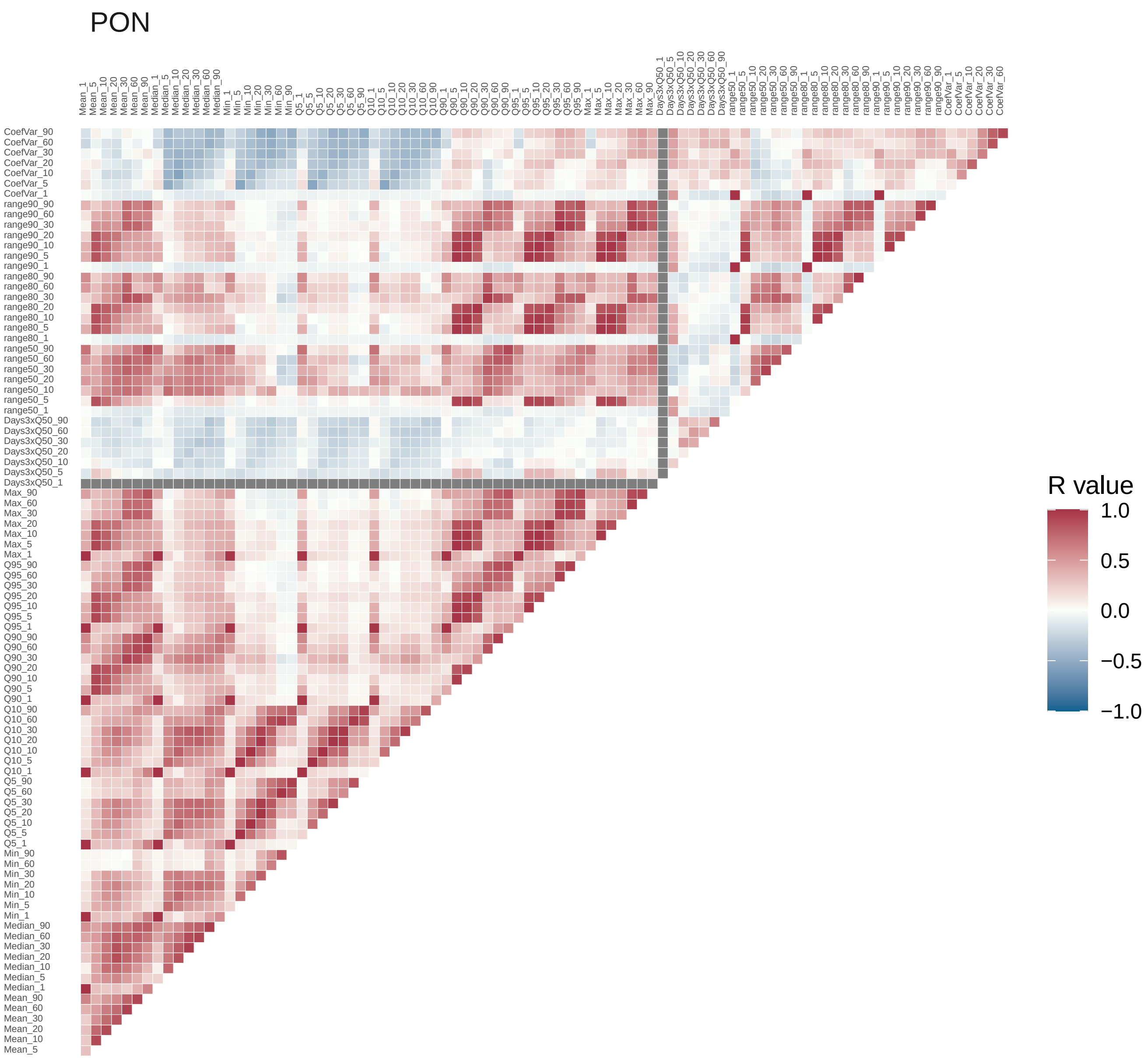
PON
R value
1.0
0.5
0.0
−0.5
−1.0

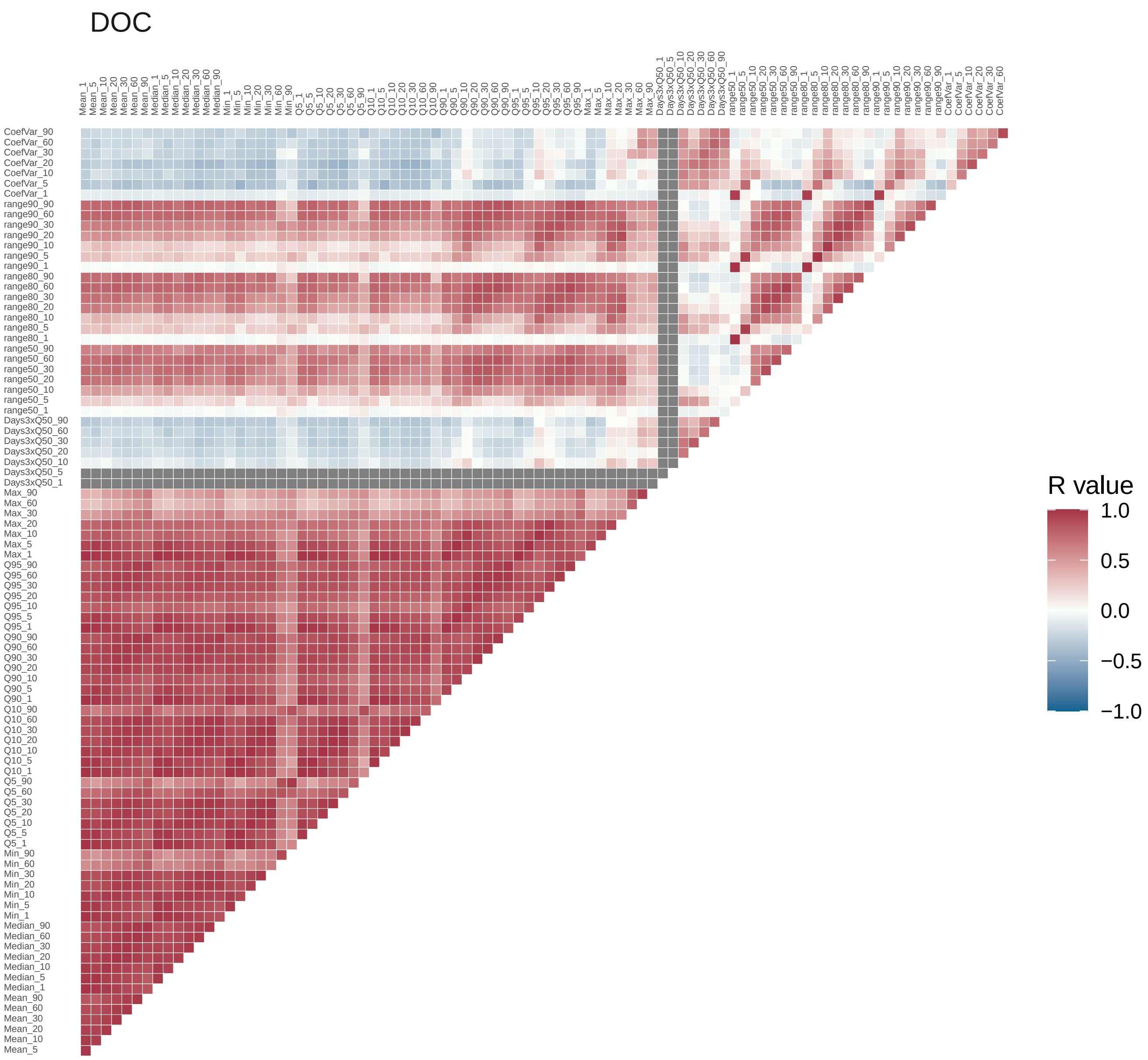
DOC
R value
1.0
0.5
0.0
−0.5
−1.0
