# Supplementary figures and images for: What do macroinvertebrate indices measure? Stressor‐specific stream macroinvertebrate indices can be confounded by other stressors
Source: Freshw Biol. 2023 May 17;68(8):1330–45. doi: 10.1111/fwb.14106 (PMC10952762; doi:10.1111/fwb.14106)

# 1 Day

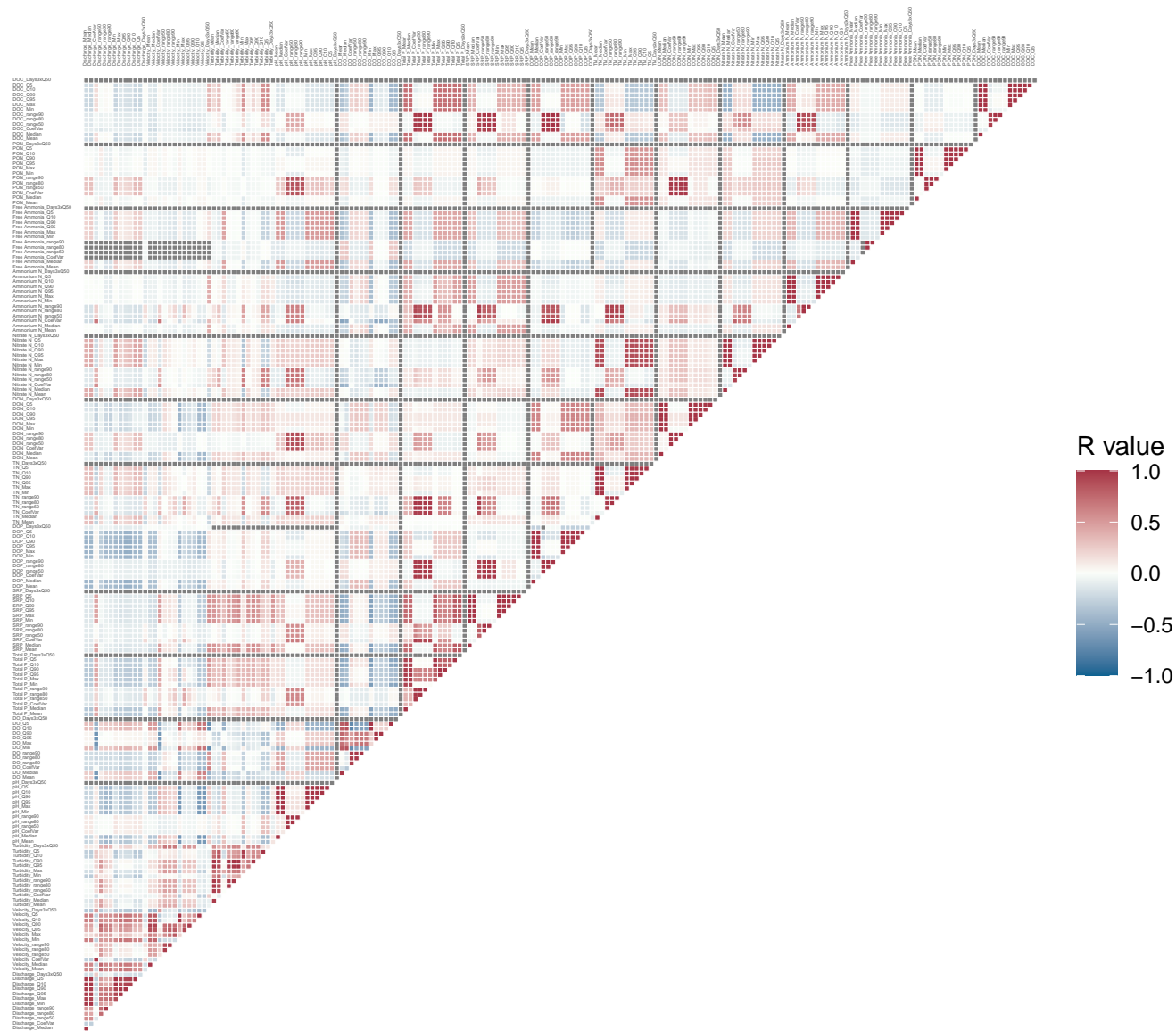

5 Days

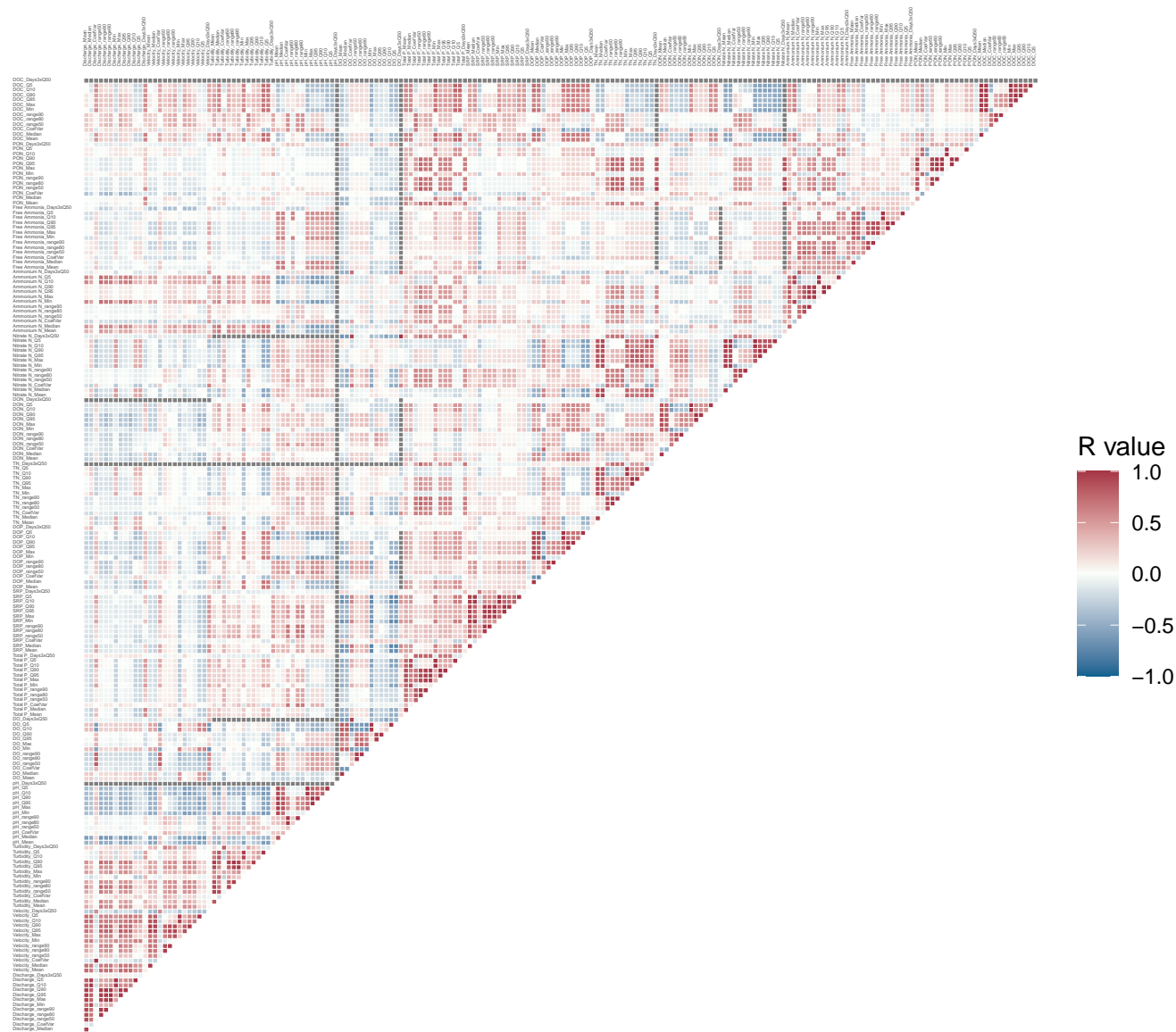

10 Days

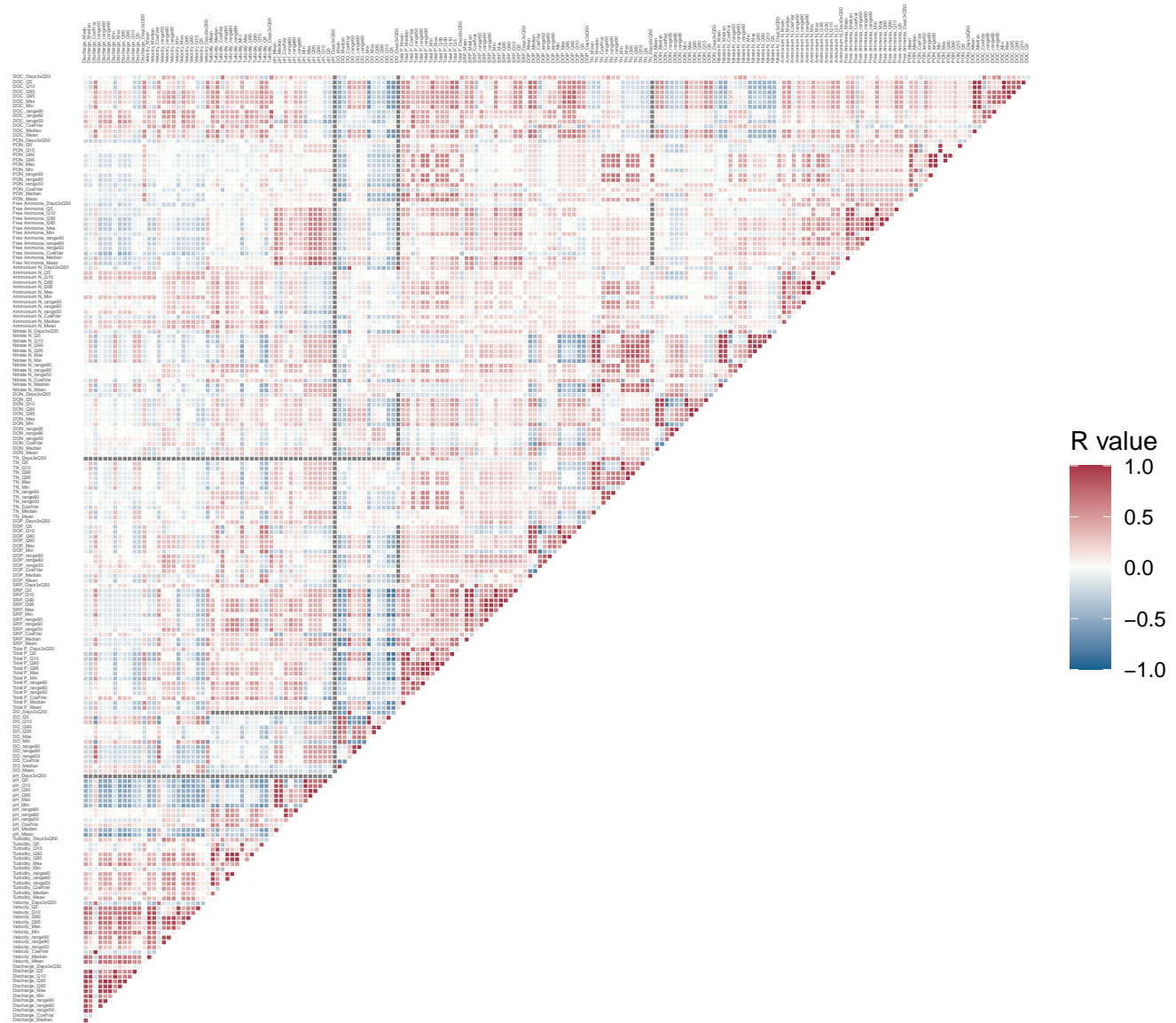

# 20 Days

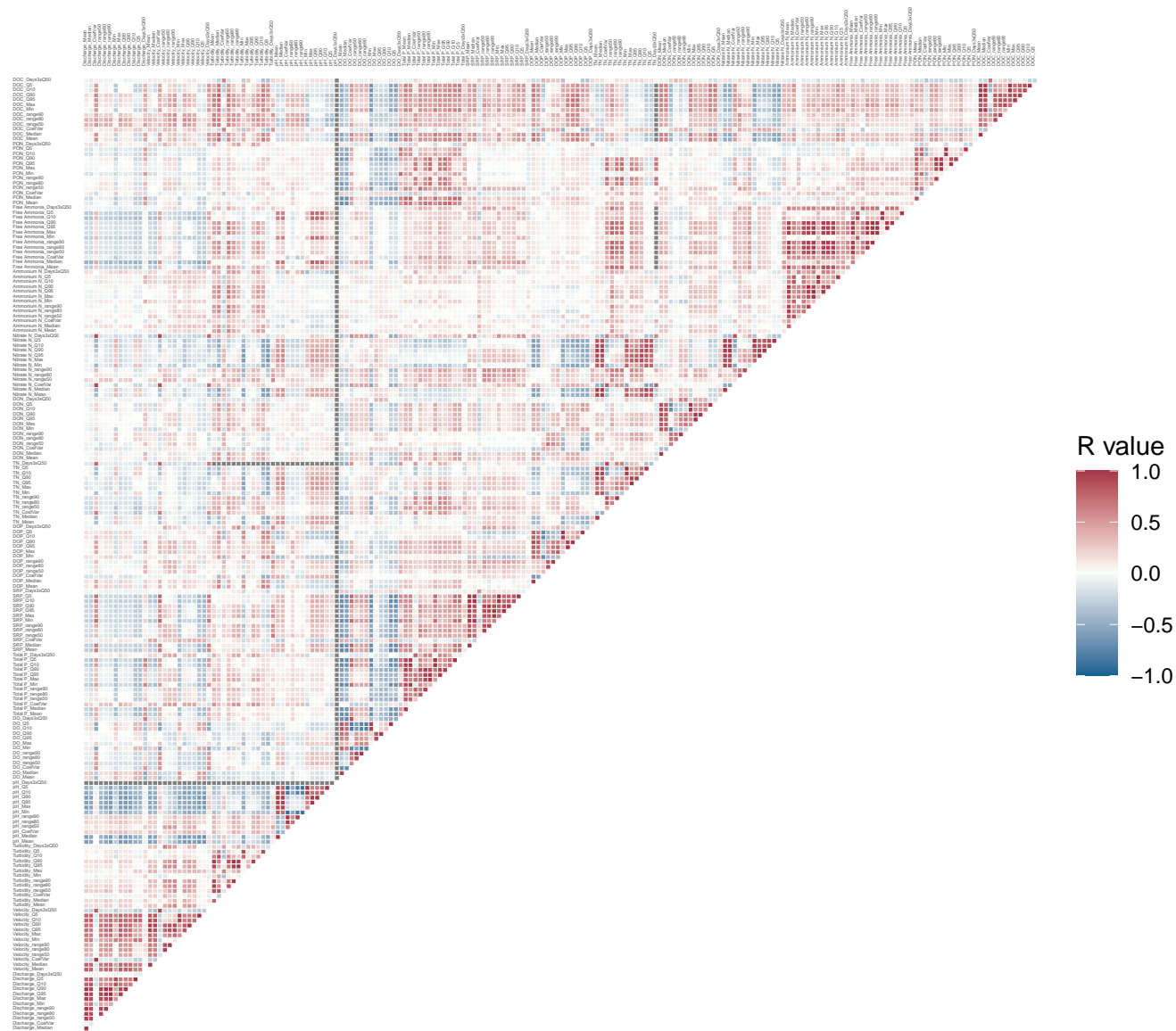

30 Days

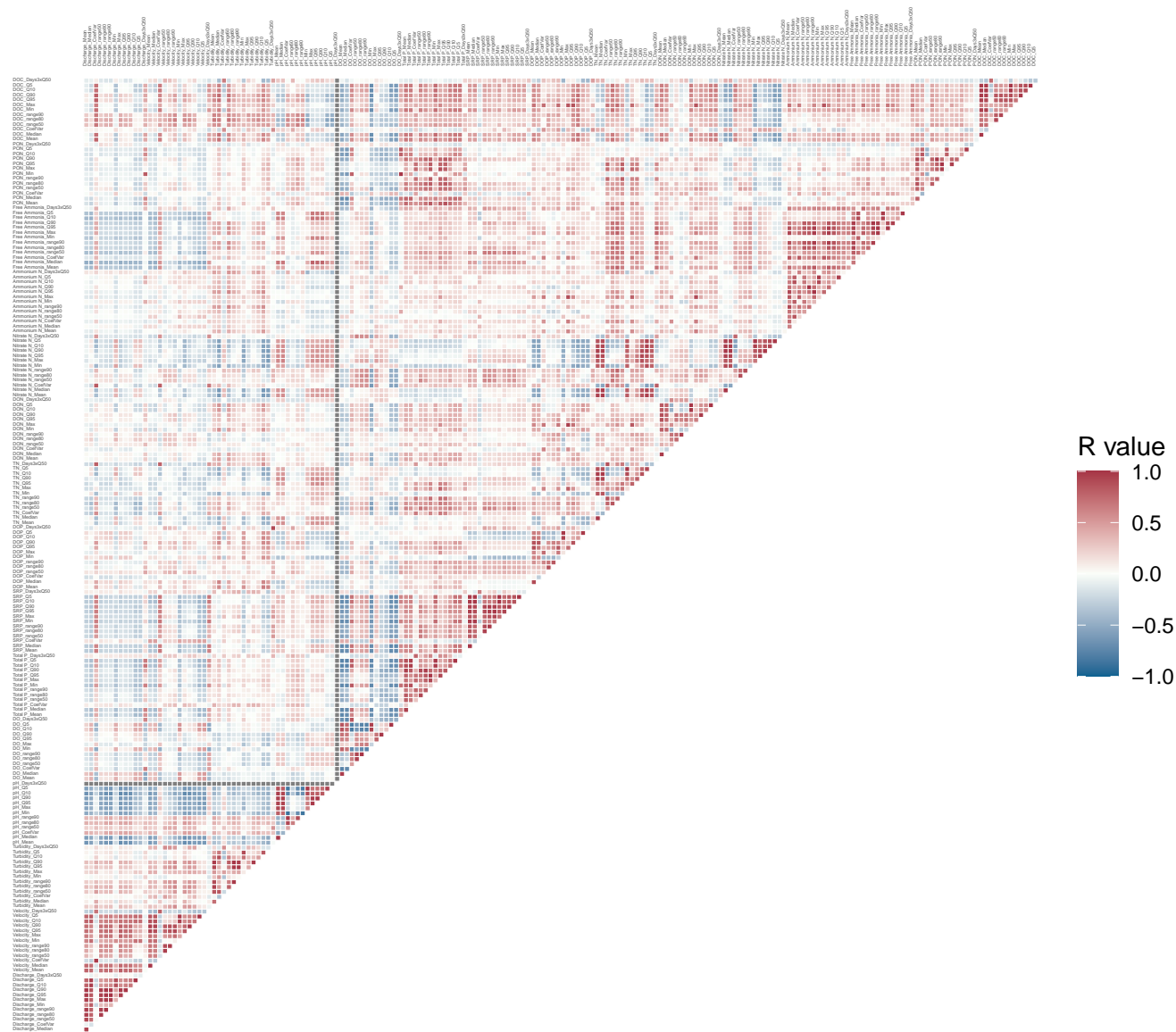

60 Days

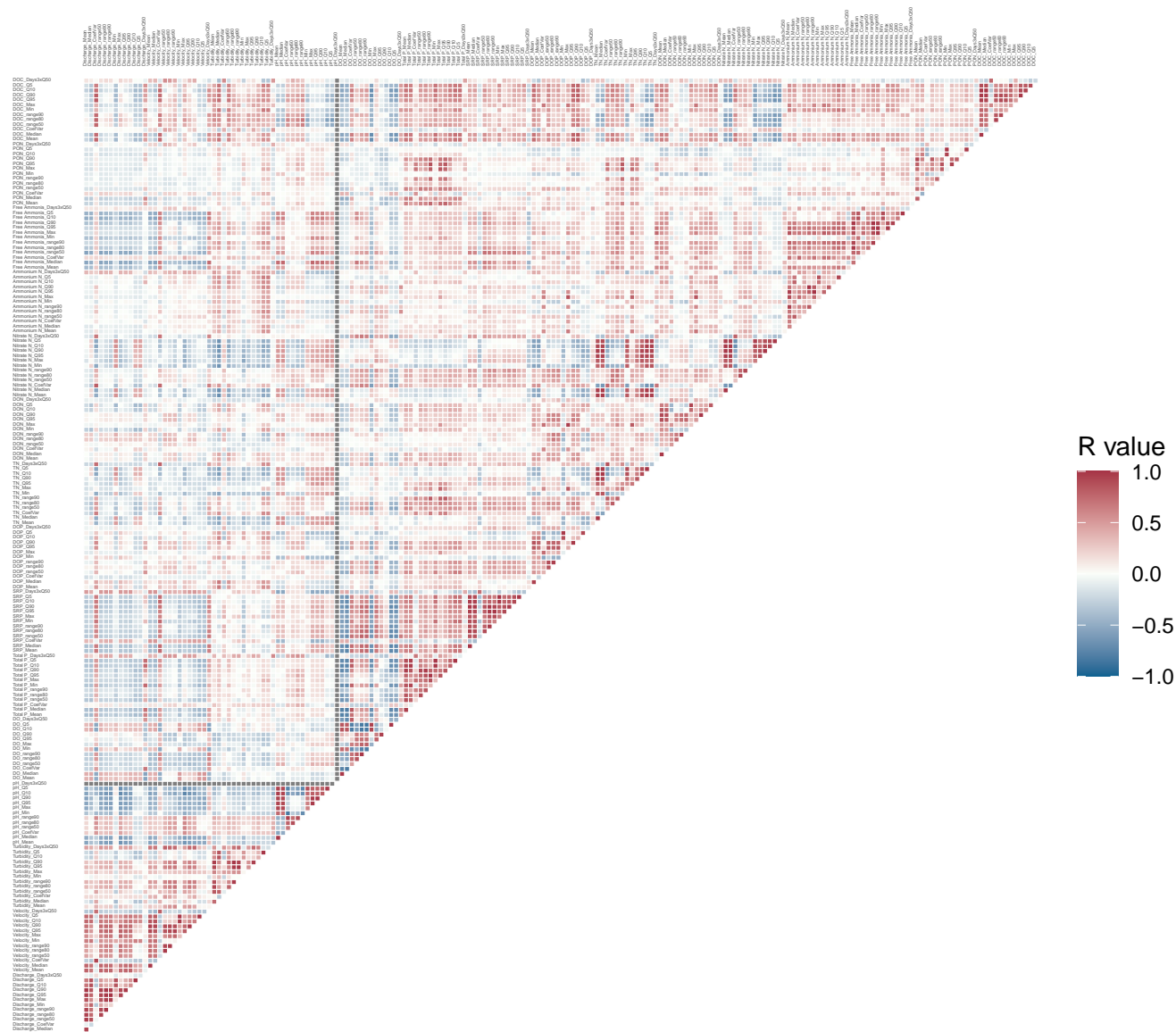

# 90 Days

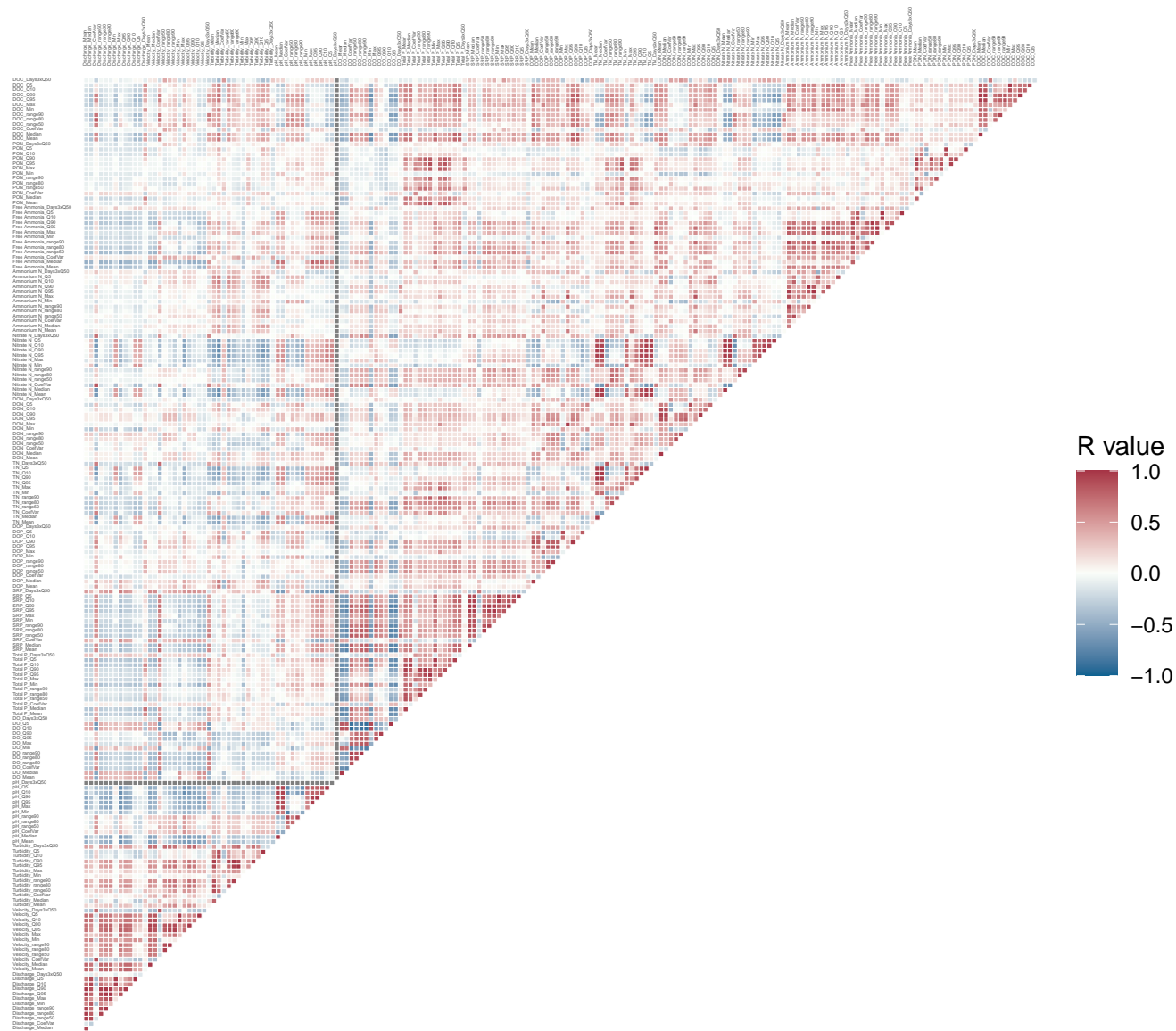

Supplement: Supplementary file 2 — Figure S2. [file FWB-68-1330-s003.pdf]
