# Supplementary material for: What do macroinvertebrate indices measure? Stressor‐specific stream macroinvertebrate indices can be confounded by other stressors
Source: Freshw Biol. 2023 May 17;68(8):1330–45. doi: 10.1111/fwb.14106 (PMC10952762; doi:10.1111/fwb.14106)
Supplement: Supplementary file 3 — Figure S3. [file FWB-68-1330-s002.docx]

Figure S3 Influence of observation period on a) mean, b) Q_5_, c) coefficient of variation, and d) range of central 90 % of observations of dissolved oxygen saturation. Each line represents an individual observation period at one site, with multiple observation periods in each site.
