# Supplementary material for: What do macroinvertebrate indices measure? Stressor‐specific stream macroinvertebrate indices can be confounded by other stressors
Source: Freshw Biol. 2023 May 17;68(8):1330–45. doi: 10.1111/fwb.14106 (PMC10952762; doi:10.1111/fwb.14106)
Supplement: Supplementary file 4 — Table S1. [file FWB-68-1330-s001.docx]

**Supplementary Information**

**Table S1. Biological indices**

| **Index** | **Name** | **Putative stressor and determinand** | **Description** | **Formula** | **Reference** |
| --- | --- | --- | --- | --- | --- |
| Log_10_ Total Abundance |  | None | Count of all macroinvertebrate individuals | Log_10_(abundance all taxa) |  |
| NTAXA_WHPT_ | Number of Taxa | General degradation - None | Number of scoring taxa following the Walley, Hawkes, Paisley and Trigg modification to the BMWP (1978) system. | N_WHPT_ | [Paisley et al. 2014](#_ENREF_29).  River Res. Appl. 30**:** 887-904. |
| ASPT_WHPT_ | Average Score Per Taxon | Organic pollution – dissolved oxygen | Average abundance weighted sensitivity to organic pollution score of taxa present. | Σ abundance weighted WHPT scores  n_WHPT_ | [Paisley et al. 2014](#_ENREF_29).  River Res. Appl. 30**:** 887-904. |
| LIFE | Lotic-invertebrate Index for Flow Evaluation | Low flows – discharge | Average abundance weighted LIFE flow score of taxa present. | Σ abundance weighted flow scores  n_LIFE_ | Extence et al. 1999.  Regul. Rivers-Res. Manage. 15**:** 543-574. |
| CoFSI | Combined Fine Sediment Index | Fine sediment – turbidity | Combined average organic sediment and total sediment sensitivity scores of taxa present. | 0.349.Σ organic sediment score + 0.569.Σ total sediment score - 6.8  n_CoFSI_ n_CoFSI_ | Murphy et al. 2015.  Freshw. Biol. 60**:** 2019-2036. |
| PSI | Proportion of Sediment-sensitive Invertebrates | Fine sediment – turbidity | Sum of abundance weighted sensitivity scores for fine sediment sensitive taxa as a percentage of the sum of abundance weighted scores for all taxa present | Σ abundance weighted scores for groups A & B . 100  Σ abundance weighted scores for groups A, B, C & D | Extence et al. 2013.  River Res. Applic. 29**:** 17-55 |
| E-PSI | Empirically-weighted Proportion of Sediment-sensitive Invertebrates | Fine sediment – turbidity | Sum of products of sensitivity score and log abundance category of fine sediment sensitive taxa as a percentage of the sensitivity score and log abundance category of all taxa | Σ (scores for group M . Log_10_ cat) .100  Σ (scores for group N . Log_10_ cat) | Turley et al. 2016.  Ecol. Ind. 70**:** 151-165. |
| TRPI | Total Reactive Phosphorus Index | Eutrophication – soluble reactive phosphorus, dissolved organic phosphorus | Sum of abundance weighted scores for total reactive phosphorus sensitive taxa as a percentage of the sum of abundance weighted scores for all taxa present | Σ abundance weighted scores for sensitive groups (A, B) . 100  Σ abundance weighted scores for all groups (A, B, C, D) | Everall et al. 2019.  Ecol. Ind. 107: 105619 |
| SPEAR_pesticide_ | SPEcies At Risk of pesticides | Pesticides – (not measured) | Abundance of pesticide sensitive taxa as a percentage of total abundance. | Σ log_10_ (abundance of sensitive taxa +1) . 100  Σ log_10_ (abundance of all taxa +1) | Beketov, et al. 2009.  Environ. Pollut. 157**:** 1841-1848 |

NB The macroinvertebrate taxa which are used to produce a score and the scores for those taxa vary among the indices. For full lists of scoring taxa and scores see original publications.

**Table S2.** **Hydrochemical determinands, summary statistics and time intervals used**

| **Determinands** | **Summary statistics** | **Time intervals** |
| --- | --- | --- |
| Discharge | Mean | 1 day |
| Velocity | Median | 5 days |
| Turbidity | Coefficient of Variation | 10 days |
| % dissolved oxygen (DO) | Range encompassing median ±25% | 20 days |
| pH | Range encompassing median ±40% | 30 days |
| Total phosphorus (TP) | Range encompassing median ±45% | 60 days |
| Soluble reactive phosphorus (SRP) | Minimum | 90 days |
| Dissolved organic P (DOP), | Maximum |  |
| Total nitrogen (TN), | Q_95_ |  |
| Nitrate nitrogen (NO_3_-N), | Q_90_ |  |
| Total ammonium (NH_4_-N), | Q_10_ |  |
| Free ammonia (NH_3_-N), | Q_5_ |  |
| Dissolved organic nitrogen (DON) | Number of days ≥3 times Q_50_ |  |
| Particulate organic nitrogen (PON) |  |  |
| Dissolved organic carbon (DOC, measured as non-purgeable organic carbon (NPOC) |  |  |

Table S3 Details of Generalized Linear Models used to assess correlation among biotic indices and between determinands and indices, where the two independent samples (random) were nested within sampling occasion (random) which was nested within site.

| **Factor** | **df** |
| --- | --- |
| Index /Determinand | 1 |
| Site | 7 |
| Index * Site | 7 |
| Occasion (Site) | 52 |
| Replicate (Occasion) | 8 |
| Error | 44 |
| Total | 119 |

**Table S4. Results of pairwise Pearson correlations among biological indices across all sites.**

R values are in bold where correlations were significant: a significant influence of site on the slope of the relationship (established by glm) indicated by * p ≤ 0.05, ** p ≤ 0.01, *** p ≤ 0.001.

|  | NTAXA | ABUNDANCE | TRPI^†^ | PSI | EPSI | coFSI | LIFE | SPEAR | ASPT |
| --- | --- | --- | --- | --- | --- | --- | --- | --- | --- |
| NTAXA |  | ** |  | ** | * |  | * | * |  |
| ABUNDANCE | -0.168 |  |  |  | ** |  |  |  |  |
| TRPI^†^ | 0.162 | -0.013 |  |  |  |  |  |  |  |
| PSI | **0.338** | **-0.236** | **0.447** |  | *** | *** |  |  | * |
| EPSI | **0.301** | **-0.192** | **0.460** | **0.610** |  | *** | ** | * | *** |
| coFSI | **0.222** | **-0.185** | **0.381** | **0.780** | **0.720** |  |  |  |  |
| LIFE | **0.348** | **-0.261** | **0.377** | **0.942** | **0.533** | **0.711** |  | * | ** |
| SPEAR | **0.392** | **-0.303** | **0.541** | **0.690** | **0.569** | **0.635** | **0.676** |  | * |
| ASPT | **0.521** | **-0.410** | **0.403** | **0.816** | **0.744** | **0.772** | **0.822** | **0.783** |  |

^†^ The TRPI index cannot be calculated using macroinvertebrate samples collected in summer, hence relationships were established on fewer samples (n = 76) compared with the other indices (n = 120).

Figure S1 Correlation matrices showing Pearson correlation coefficients of pairwise combinations of summary statistics within determinands across all time periods. (see file FigS1.pdf). Panels show heat maps, where intensity of colour reflects the correlation coefficient for each pairwise comparison (red positive correlation, blue negative correlation). Panels are arranged by determinand. For each determinand, pairwise comparisons are arranged by summary statistic with increasing time period (1 day to 90 days) over which they were calculated within each summary statistic. The figure shows how different aspects of variation within each determinand are related to one another and how these vary over time.

Figure S2 Correlation matrices showing Pearson correlation coefficients of pairwise combinations of summary statistics within time periods across all determinands. (see file FigS2.pdf). Panels show heat maps, where intensity of colour reflects the correlation coefficient for each pairwise comparison (red positive correlation, blue negative correlation). Panels are arranged by time period, 1 day to 90 days, over which the summary statistics were calculated. For each time period, pairwise comparisons are arranged by determinand, with different summary statistics within each determinand. The figure shows how different determinands are related to one another and, by comparison across the panels how these relationships vary with increasing time periods over which the summary statistics were calculated.
